# Circular RNA DNAH14 molecular mechanism in an experimental model of hepatocellular carcinoma treated with Cobalt chloride to mimic the hypoxia-like response of transcatheter arterial chemoembolization

**DOI:** 10.1038/s41598-024-52578-3

**Published:** 2024-01-23

**Authors:** Qiuling Liao, Weiping Xia, Jiawen Chen, Kangning Wang, Enhua Xiao

**Affiliations:** 1 Department of Radiology, The Second Xiangya Hospital of Central South University, No. 139, Renmin Middle Road, Changsha City, 410011 Hunan Province China; 2 Department of Urology Surgery, Xiangya Hospital Central South University, No. 87 Xiangya Road, Kaifu District, Changsha City, 410008 Hunan Province China

**Keywords:** Cell biology, Oncology, Cancer, Tumour biomarkers

## Abstract

Transcatheter arterial chemoembolization (TACE) is the primary local treatment for patients with unresectable hepatocellular carcinoma (HCC). Numerous studies have demonstrated the pivotal role of circular RNAs (circRNAs) in TACE efficacy. This study aimed to investigate the function of circular RNA DNAH14 (circDNAH14) in TACE for HCC and to elucidate its molecular mechanisms. To simulate hypoxia conditions experienced during TACE, HCC cells were treated with cobalt chloride. The expression levels of circDNAH14, microRNA-508-3p (miR-508-3p), and Prothymosin Alpha (PTMA) were modulated via transfection for knockdown or overexpression. Cell Counting Kit-8 and 5-ethynyl-2′-deoxyuridine assays, flow cytometry, and Transwell assays, along with epithelial-mesenchymal transition (EMT) evaluations, were employed to assess cell proliferation, apoptosis, invasion, migration, and EMT. The results indicated that hypoxia treatment downregulated the expression of circDNAH14 and PTMA while upregulating miR-508-3p. Such treatment suppressed HCC cell proliferation, invasion, migration, and EMT, and induced apoptosis. Knockdown of circDNAH14 or PTMA intensified the suppressive effects of hypoxia on the malignant behaviors of HCC cells. Conversely, upregulation of miR-508-3p or PTMA mitigated the effects of circDNAH14 overexpression and knockdown, respectively. Mechanistically, circDNAH14 was found to competitively bind to miR-508-3p, thereby regulating PTMA expression. In vivo, nude mouse xenograft experiments demonstrated that circDNAH14 knockdown augmented the hypoxia-induced suppression of HCC tumor growth. In conclusion, circDNAH14 mitigates the suppressive effects of hypoxia on HCC, both in vitro and in vivo, by competitively binding to miR-508-3p and regulating PTMA expression.

## Introduction

Hepatocellular carcinoma (HCC) is the major reason for cancer-linked deaths worldwide^1^. Because most patients are in the advanced stage when being diagnosed, the 5-year survival rate of HCC patients and the prognosis are poor^2^. At present, chemotherapy, drug therapy, surgical resection, and liver transplantation are frequently applied for HCC clinical treatment^3^. However, not all HCC patients can be surgically treated^4^.

Transcatheter arterial chemoembolization (TACE) is the delivery of chemotherapy drugs and embolic agents directly into the blood vessels of the tumor through intubation, thereby cutting off the blood supply to the tumor and applying chemotherapy drugs locally. This method can significantly reduce the blood supply to the tumor, causing tumor cell necrosis, while reducing systemic side effects^5^. While surgical resection and liver transplantation are seen as potentially radical treatments in early HCC, many patients are already in the middle or advanced stages of diagnosis and are unable to receive these treatments^6^. As a result, TACE has become the primary topical treatment for patients with mid-stage HCC, especially for those who are not candidates for surgical treatment or are waiting for a liver transplant^7^. However, there is individual variation in the efficacy of TACE^8^. In addition, the success of TACE treatment is often affected by the effect of iodized oil deposition^9^. This study focused on exploring the potential mechanisms that influence the effect of TACE therapy, providing strong data support for understanding the genetic changes in TACE therapy.

Circular RNA (circRNA) is a kind of endogenous non-coding RNA with a closed-loop structure. It has recently been considered a key modulator of gene expression and pathological networks^10^. CircRNAs participate in HCC development and progression^11,12^. CircDNAH14 has been found to be elevated in plasma exosomes of HCC patients^13^. Nevertheless, the role of circDNAH14 in HCC remains unclear.

In this study, it is hypothesized that circDNAH14 may be involved in the regulation of TACE in the treatment of HCC. In vitro hypoxic HCC cell model was established to simulate the anoxic process of TACE. Functional rescue experiments confirmed that circDNAH14 affected the proliferation, invasion, migration, apoptosis and epithelial-mesenchymal transition (EMT) processes of hypoxic HCC cells by regulating the miR-508-3p/prothymosin α (PTMA) axis.

## Materials and methods

### Cell culture

Human HCC cell lines PLC/PRF/5, HepG2, and SMMC7721 (Cell Bank of Chinese Academy of Sciences, Shanghai, China) were cultured in Roswell Park Memorial Institute-1640 medium covering 10% fetal bovine serum (Excell Bio, China) and 1% penicillin–streptomycin (Invitrogen) and placed in an incubator (37 °C, 5%CO_2_). To simulate TACE hypoxia, cells were treated with 150 μM cobalt chloride for 24 h^14^. Since the expression of circDNAH14 in SMMC-7721 was the lowest after hypoxic treatment, SMMC-7721 was selected for follow-up study.

### Cell transfection

Specially designed small interfering RNAs (siRNAs) and overexpressed plasmid (pcDNA 3.1) were used to target circDNAH14 (si-circDNAH14: 5′-ATTTTCTGATTTCCGCCAGTT-3′, oe-circDNAH14) and PTMA (si-PTMA: 5′-GAGGAAAAAAGAACCAAAACTTC-3′). These siRNA and overexpressed plasmids were custom-designed by GeneChem (Shanghai, China) to meet specific requirements. In addition, mimic and inhibitor of miR-508-3p and corresponding negative controls (mimic/inhibitor NC) were provided by RIBOBIO (Guangzhou, China). With 70% cell confluence, transfection was done in hypoxic SMMC-7721 cells using Lipofectamine 3000 (Invitrogen, Eugene, OR, USA). After 48 h, the changes of RNA were detected by real-time reverse transcriptase-polymerase chain reaction (RT-qPCR). Protein changes were evaluated by western blot. Information on overexpressed plasmids is shown in Supplementary Figs. 1 and 2.

### Cell counting kit (CCK)-8

Transfected or hypoxic SMMC-7721 cells were seeded in a 96-well plate at 3000 cells/well, and 10 µL CCK-8 solution (Dojindo Molecular Technologies, Kumamoto, Japan) was added at 0, 24, 48, and 72 h. After 2 h, the absorbance at 450 nm was read on the EPOCH™ spectrophotometer (Epoch, BioTek Instruments, Inc., Winooski, VT, USA)^15^.

### 5-ethynyl-2'-deoxyuridine (EdU) assay

Transfected or hypoxic SMMC7721 cells were plated in a 96-well plate at 1 × 10^4^/well and detected for proliferation rate using Cell-Light EdU DNA Cell Proliferation Kit (RiboBio, Guangzhou, China). When the cells adhered to the wall, they were then incubated with 50 μL EdU-A solution. Two hours later, the cells were immobilized with the Apollo dye solution. Then, Hoechst 33,342 solution was added, and cell proliferation was evaluated by calculating the ratio of EdU-stained cells to Hoechst-stained cells. Images were taken under a fluorescence microscope (Olympus, Tokyo, Japan)^16^.

### Flow cytometry assessment of apoptosis

Analysis of SMMC7721 cell apoptosis was done using the Alexa Fluor 488 Annexin V Kit (Invitrogen). In brief, SMMC7721 cells (1 × 10^6^ cells/mL) were washed with phosphate buffered saline (PBS) and centrifuged, and the pellet was resuspended in an Annexin binding buffer and incubated with annexin V and propidium iodide working solutions. Apoptosis was determined on a flow cytometer (BD Biosciences, San Jose, CA, USA) and evaluated by FlowJo software (Tree Star, San Carlos, CA)^17^.

### Transwell assays

For migration assay, cells after starvation were added to the upper chamber of a 24-well plate (Shanghai Jrdun Biotechnology, China). For invasion assay, Matrigel (1:4, Qcbio, Shanghai, China) was coated on the surface of the upper chamber membrane. Cells (approximately 1 × 10^5^ cells per well) were maintained in a serum-free medium, and 600 μL Roswell Park Memorial Institute-1640 medium containing 20% fetal bovine serum was added to the lower chamber. After 48 h, cells in the chamber were fixed with 4% paraformaldehyde and stained with 0.5% crystal violet. The invading or migrating cells were observed and imaged by light microscopy (Olympus, Tokyo, Japan)^18^.

### RT-qPCR

Isolation of Total RNA from tissues and cell lines was done using AG RNAex Pro RNA extraction kit AG21102 (Accurate Biotechnology (Hunan) Co., Ltd., Changsha, China), followed by measurements of RNA purity and concentration via NanoDrop™ 2000 spectrophotometer (Thermo Fisher Scientific, Waltham, MA, USA). Complementary DNA (cDNA) was generated using Evo M-MLVPrimeScript RT Master Mix reverse transcription Kit with gDNA Clean for qPCR AG11728 (Accurate Biotechnology), and evaluated by QuantStudioTM 5 real-time PCR instrument(96-Well 0.2 ml Block) (Thermo Fisher Scientific, Waltham, MA, USA). Real-time quantitative PCR was performed via SYBR Green Premix Pro Taq HS qPCR Kit (Rox Plus) AG11718 (Accurate Biotechnology). Glyceraldehyde-3-phosphate dehydrogenase (GAPDH) and U6 were loading control genes. The selection of GAPDH and U6 as loading control genes was based on their wide application and expression stability in RNA analysis. Gene expression was measured by the 2-^ΔΔCt^ method. Primer sequences were manifested in Table 1^19^.

**Table 1:** PCR Primer sequence.

	Primer sequence (5′–3′)
CircDNAH14	F: 5′-GCATTTGTTACCTGGAAATTGA-3′
	R: 5′-CTTGGTTTCCTCCTTGTCCA-3′
MiR-508-3p	F: 5′-GTACTCCAGAGGGCGTCA-3′
	R: 5′-GCAGGGTCCGAGGTATTC-3′
GAPDH	F: 5′-CACCCACTCCTCCACCTTTG-3′ R: 5′-CCACCACCCTGTTGCTGTAG-3′
U6	F: 5′-CTCGCTTCGGCAGCACA-3′
	R: 5′-AACGCTTCACGAATTTGCGT-3′

F, forward; R, reverse.

### Western blot

Radioimmunoprecipitation assay lysis buffer (China Institute of Biotechnology, Beyotime) was added to tissues and SMMC-7721 cells to prepare protein samples. Bicinchoninic acid protein quantification kits (Bio-Rad) were utilized for detection of protein concentration. The lysate was mixed with the loading buffer and denatured in boiling water. Next, the same amount of sample was separated by sodium dodecyl sulfate polyacrylamide gel electrophoresis and transferred to the polyvinylidene fluoride membrane. After blocking the membrane with 5% skim milk at room temperature for 30 min, it was placed with the primary antibody at 4 °C overnight. Next, the membrane and horseradish peroxidase-coupled secondary antibody (ab205718; Abcam) were incubated at room temperature for 1 h. Finally, protein bands were developed using enhanced chemiluminescence Blot substrates (Promega). The antibody information was as follows: PTMA (YN2871, immunoway), E-cadherin (20874-1-AP), N-cadherin (22018-1-AP) (Proteintech), Snail (ab53519), GAPDH (ab8245) (Abcam)^20^.

### Tumor xenograft

Twelve BALB/c nude mice (male, 4–6 weeks old, weighing 18–20 g) were purchased from China Hunan Slack Jingda Laboratory Animal Co., Ltd, and fed under specific pathogen-free conditions and a 12-h light cycle in the animal cages. The animals were divided into the following 4 groups: Normoxia group, Hypoxia group, si-NC group, and si-circDNAH14 group. A suspension of hypoxic SMMC-7721 cells (2 × 10^7^ cells, stably knocked down or not knocked down circDNAH14) or normoxia control SMMC-7721 cells (2 × 10^7^ cells) was injected subcutaneously into each mouse. Tumor volume was measured every 5 days using vernier calipers. Volume formula = (length × width^2^) × 0.5. Finally, euthanasia of the mice was conducted and tumor tissues were collected for subsequent analysis.

### Dual luciferase reporter experiment

Starbase bioinformatics website (https://rnasysu.com/encori/) predicted targeted binding sites between miR-508-3p and circDNAH14 or PTMA. circDNAH14 and PTMA 3'-untranslated region (UTR) containing presumed miR-508-3p binding sites were amplified by PCR from human genomic DNA. The DNA fragment was cloned into the pmiR-RB-REPORT vector (RiboBio) and named wild type (WT)-circDNAH14/PTMA. miR-508-3p binding sites in circDNAH14 and PTMA 3'-UTR were mutated to obtain mutant luciferase reporter vectors. The above luciferase reporter vector and miR-508-3p mimic or mimic NC were co-transfected into SMMC-7721 cells using Lipofectamine 3000. Luciferase activity was measured 24 h after transfection and reported according to the manufacturer's protocol (Promega, E2920). Renilla luciferase activity was normalized against firefly luciferase activity and reported as a percentage of the control group.

### Radioimmunoprecipitation (RIP) assay

The crude cell lysate was incubated with the RIP buffer. To prepare the RIP buffer, magnetic beads coupled with human anti-AGO2 or mouse immunoglobulin G were added. The complex was harvested with protease K and then the immunoprecipitated RNA was isolated. RNA concentration measurements and RNA quality assessments were performed by spectrophotometers (NanoDrop, Thermo Scientific, Waltham, MA, USA). To demonstrate the presence of the binding target, purified RNA was collected and tested by RT-qPCR.

### Immunohistochemistry

Tumor tissue sections (4 μm) were deparaffinized with xylene (twice, 5 min each), and rehydrated with graded ethanol. Sections were then soaked in deionized water for 5 min and dried with absorbent paper. Antigen retrieval was performed by heating the samples in a microwave oven. After cooling, sections were treated with 3% H_2_O_2_ for 10 min, washed with PBS, and blocked with 3% bovine serum albumin for 1 h. The sections were then incubated with primary antibodies Ki-67 (ab15580, Abcam) and cleaved caspase-3 (9661, Cell Signaling Technology) overnight at 4 °C, followed by horseradish peroxidase-conjugated secondary antibody (Dako, Canada) for 1 h at 37 °C. Sections were then treated with diaminobenzidine and counterstained with hematoxylin for 45 s. Finally, the sections were washed with ultrapure water, dehydrated with gradient ethanol, dewaxed with xylene for 10 min, dried, and sealed^21^.

### Data analysis

SPSS 20.0 statistical software (IBM Corporation, USA) was utilized for data analysis. Measurement data were presented as mean ± standard deviation (SD). The two-group comparison was done using the two-tailed student t-test, and the comparison of multiple groups was carried out with one-way analysis of variance (ANOVA). After ANOVA, Tukey’s multiple comparison follow-up test was conducted. *P* < 0.05 emphasized obvious statistical meaning. All experiments in this study were biologically replicated at least three times.

### Ethical approval

All animal experiments complied with the ARRIVE guidelines and performed in accordance with the National Institutes of Health Guide for the Care and Use of Laboratory Animals. Approval of animal experiments was obtained from the Animal Research Committee of The Second Xiangya Hospital of Central South University (approval number: 2021801).

## Results

### Knockdown of circDNAH14 strengthens the inhibitory effect of hypoxia on HCC cells

**Figure 1:**
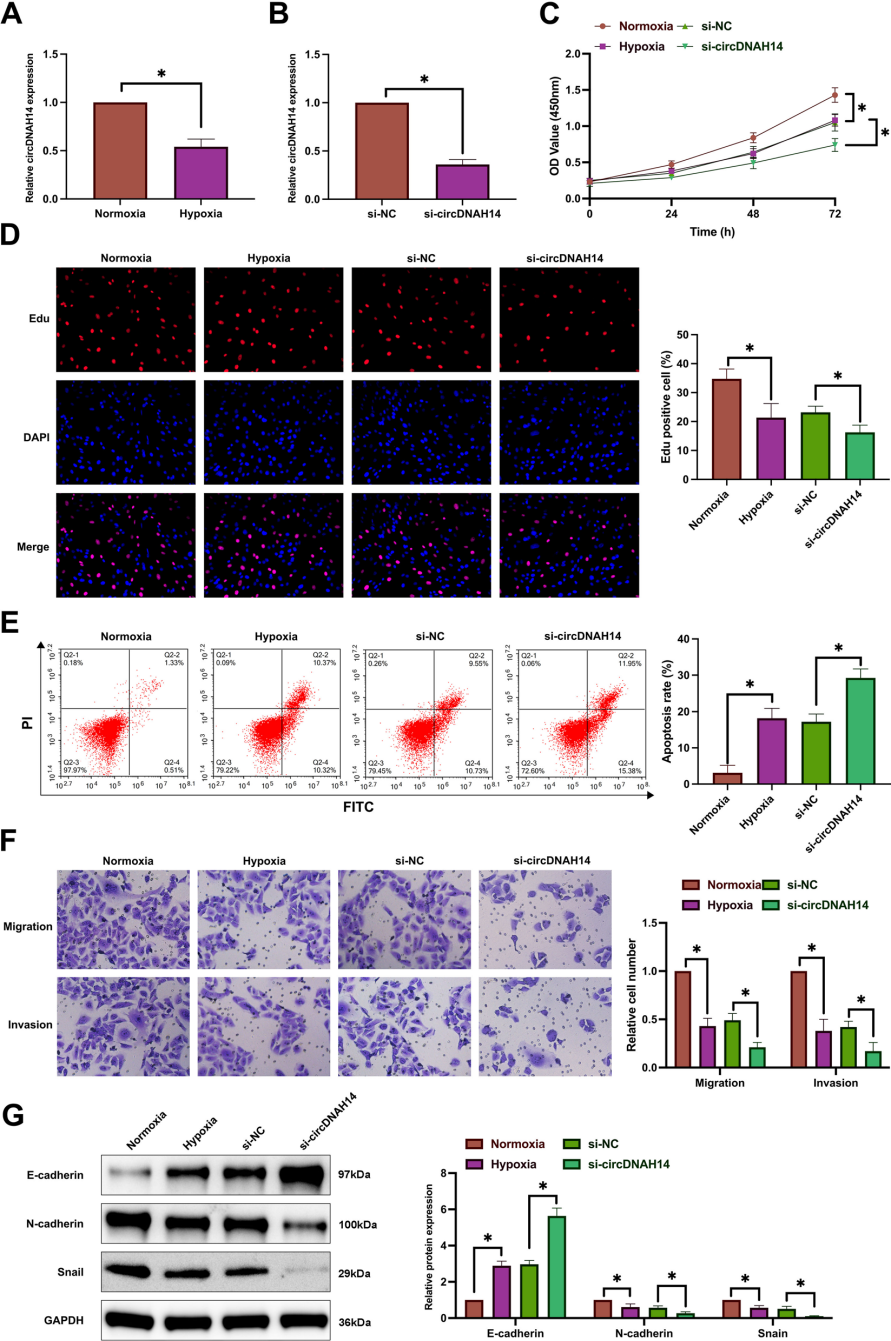
Knockdown of circDNAH14 enhances the inhibitory effect of hypoxia on HCC cells. SMMC-7721 cells were treated with hypoxia or circDNAH14 knockdown simultaneously with hypoxia. (**A**), (**B**) RT-qPCR detection of circDNAH14; (**C**) CCK-8 detection of cell proliferation; (**D**) EdU analysis detection of cell DNA replication; (**E**) Flow cytometry detection of cell apoptosis; (**F**) Transwell detection of cell invasion and migration abilities; (**G**) Western blot detection of E-cadherin, N-cadherin, and Snail in cells; Data are presented as mean ± SD (N = 3). *P < 0.05.

As shown in Fig. 1A, under hypoxic conditions, the expression of circDNAH14 was decreased. Since the expression of circDNAH14 decreased the most in hypoxic SMMC-7721 cells, SMMC-7721 cells were selected for follow-up experiments (Supplementary Fig. 3A). Subsequently, by transfecting si-circDNAH14, circDNAH14 was further down-regulated in hypoxic SMMC-7721 cells (Fig. 1B). The proliferation ability of SMMC-7721 cells was evaluated by CCK-8 and EdU experiments, and the results showed that hypoxia inhibited the proliferation of SMMC-7721 cells and the proportion of EdU-positive cells, while knockdown of circDNAH14 promoted the effect of hypoxia (Fig. 1C,D). Flow cytometry showed that hypoxia led to an increase in the apoptotic rate of SMMC-7721 cells, while knockdown of circDNAH14 further increased the apoptotic rate (Fig. 1E). The invasion and migration ability of SMMC-7721 cells were examined by Transwell assays. As shown in Fig. 1F, hypoxia reduced the invasion and migration ability of SMMC-7721 cells, which was further reduced after knockdown of circDNAH14. EMT-related proteins were assessed by Western blot. As shown in Fig. 1G, hypoxia increased the expression of E-cadherin and down-regulated the expression of N-cadherin and Snail in SMMC-7721 cells, and knockdown of circDNAH14 enhanced this effect. These results indicated that knockdown of circDNAH14 enhanced the effect of hypoxia on SMMC-7721 cells.

### CircDNAH14 competitively absorbs miR-508-3p

**Figure 2:**
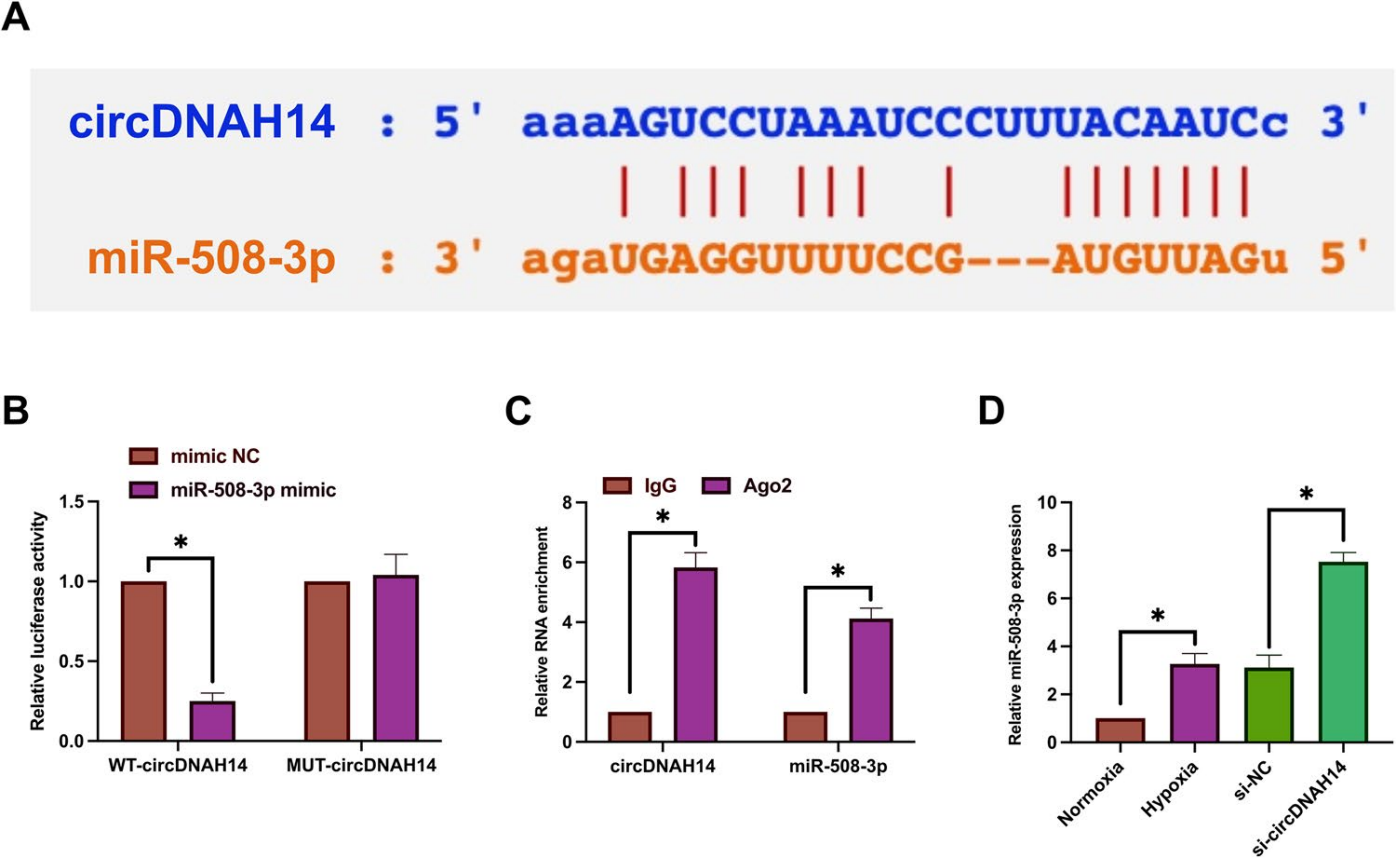
CircDNAH14 competitively absorbsmiR-508-3p. (**A**) Bioinformatics website http://starbase.sysu.edu.cn/ predicted binding sites for miR-508-3p and circDNAH14; (**B**), (**C**) Dual luciferase reporter and RIP experiments verified the targeting relationship between miR-508-3p and circDNAH14; (**D**) RT-qPCR detection of miR-508-3p expression under hypoxia induction and circDNAH14 knockdown; Data are presented as mean ± SD (N = 3). *P < 0.05.

CircRNAs compete with proteins for miRNA binding sites to exert biological effects^22^. Next, the downstream targets of circDNAH14 were explored. miR-508-3p was identified as the downstream target gene of circDNAH14. As shown in Fig. 2A,B, there were potential binding sites shared by circDNAH14 and miR-508-3p. In addition, co-transfection of WT-circDNAH14 and miR-508-3p mimic reduced the luciferase activity of SMMC-7721 cells, while co-transfection of MUT-circDNAH14 and miR-508-3p mimic had no effect on the luciferase activity. This targeting relationship was further determined by RIP experiments. The results showed that compared with IgG, circDNAH14 and miR-508-3p were significantly enriched in Ago2 magnetic beads (Fig. 2C). After hypoxia induction, the expression of miR-508-3p was significantly increased, and knockdown of circDNAH14 promoted the expression of miR-508-3p (Fig. 2D). These results suggest that circDNAH14 competitively absorbs miR-508-3p.

### Knockdown of miR-508-3p attenuates the effect of hypoxia on HCC cells

**Figure 3:**
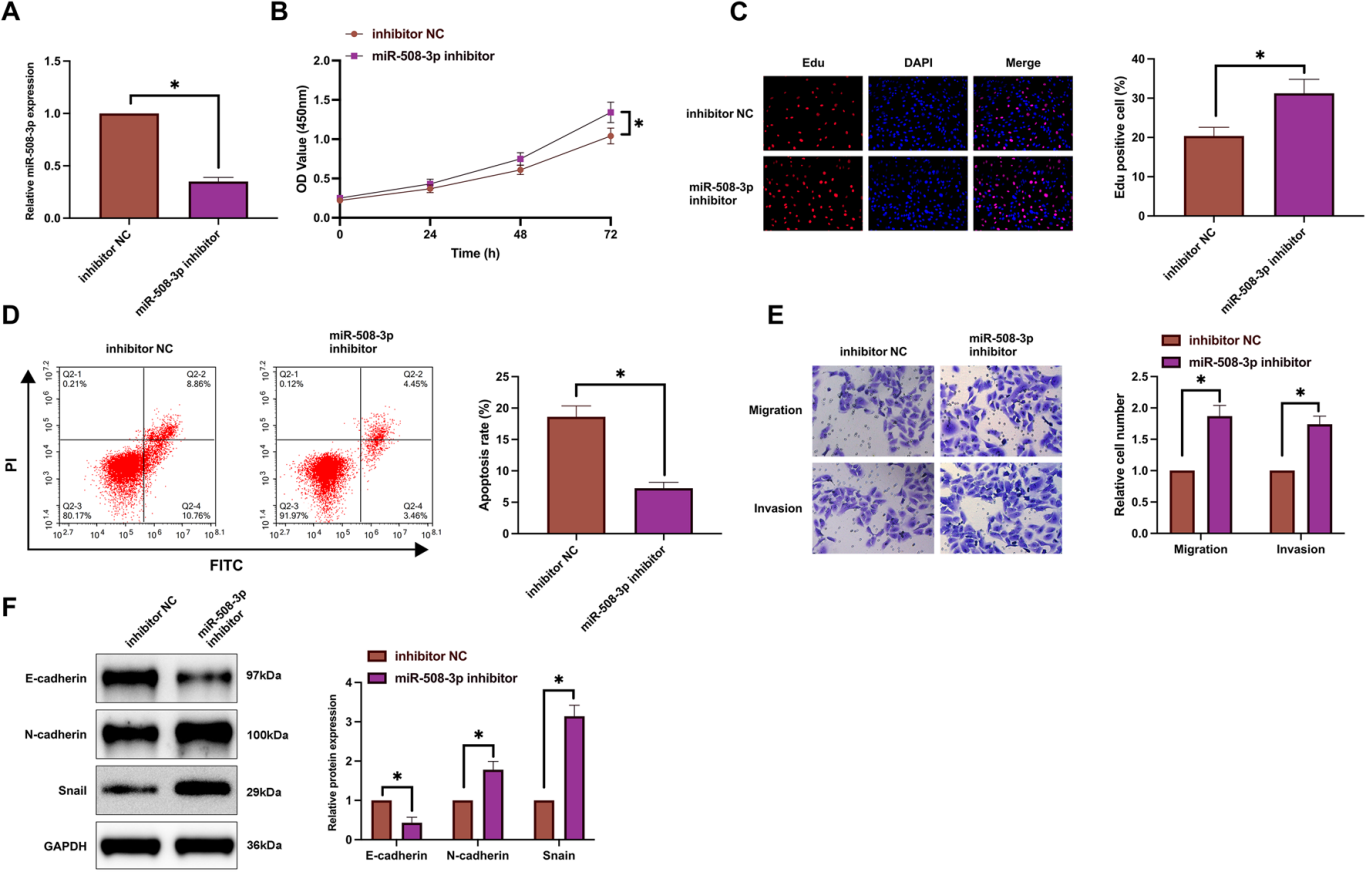
Depression of miR-508-3p reduces hypoxia-stimulated suppression of HCC cell development. MiR-508-3p in SMMC-7721 cells was silenced during hypoxia induction. (**A**) RT-qPCR evaluation of miR-508-3p expression; (**B**) CCK-8 detection of cell proliferation; (**C**) EdU analysis detection of cell DNA replication; (**D**) Flow cytometry detection of cell apoptosis; (**E**) Transwell detection of cell invasion and migration abilities; (**F**) Western blot detection of E-cadherin, N-cadherin, and Snail in cells; Data are presented as mean ± SD (N = 3). *P < 0.05.

miR-508-3p was down-regulated in hypoxic SMMC-7721 cells by transfection with miR-508-3p inhibitor (Fig. 3A). Functional experiments showed that after knockdown of miR-508-3p, the proliferation rate of hypoxic SMMC-7721 cells and the proportion of EdU-positive cells were increased (Fig. 3B, C), the apoptosis rate was reduced (Fig. 3D), invasion and migration abilities were enhanced (Fig. 3E), N-cadherin and Snail expression increased while E-cadherin expression decreased (Fig. 3F). Shortly, knockdown of miR-508-3p attenuates the inhibitory effect of hypoxia on HCC cells.

### MiR-508-3p targets PTMA

**Figure 4:**
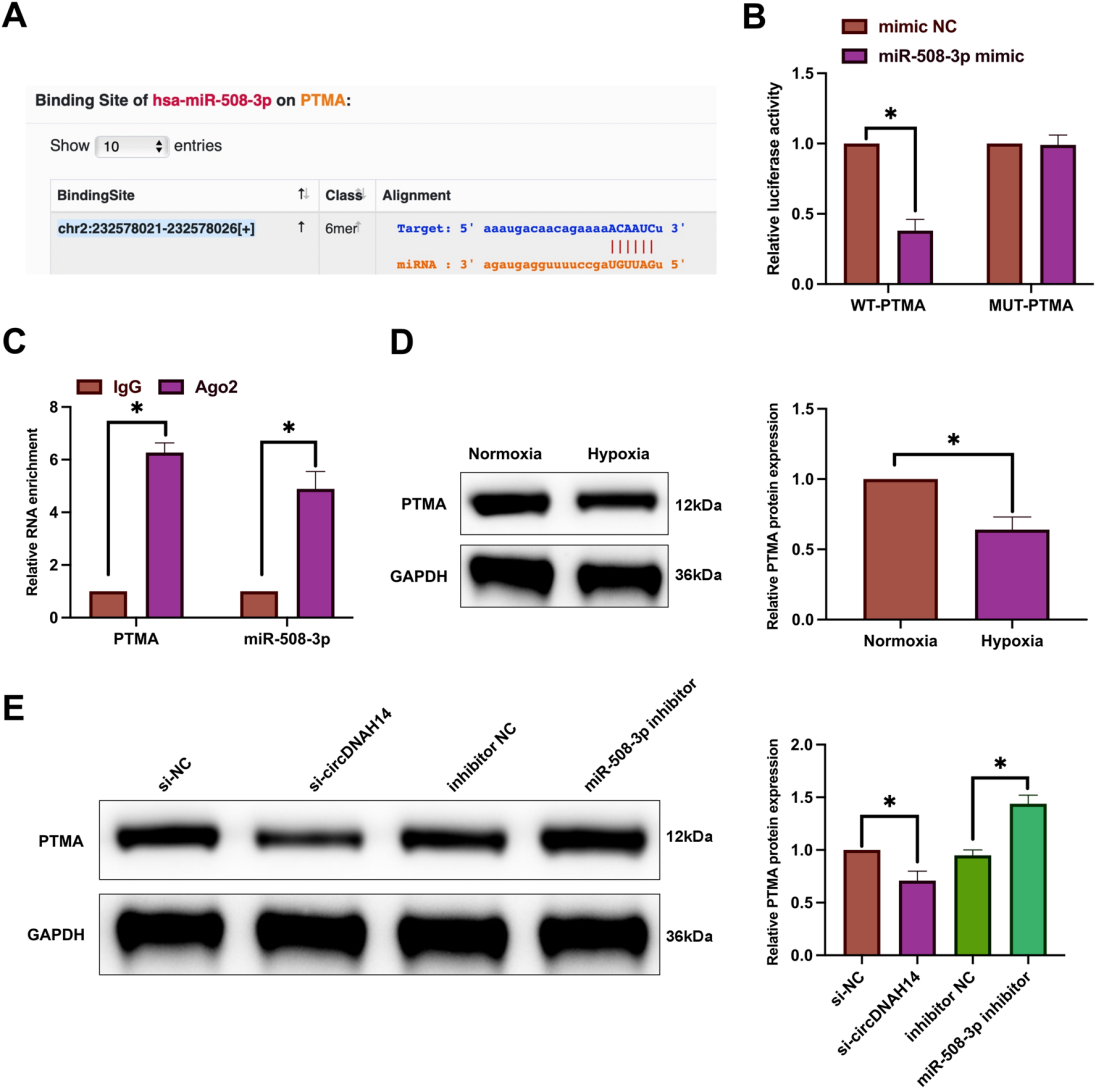
MiR-508-3p targets PTMA. (**A**) Bioinformatics website http://starbase.sysu.edu.cn/ predicted binding sites for miR-508-3p and PTMA; (**B**), (**C**): Dual luciferase reporter and RIP experiments verified the targeting relationship between miR-508-3p and PTMA; (**D**), (**E**): Western blot detection of PTMA in SMMC-7721 cells after altering circDNAH14/miR-508-3p expression; Data are presented as mean ± SD (N = 3). *P < 0.05.

Next, the target genes of miR-508-3p were explored. PTMA may be a downstream target gene of miR-508-3p. As shown in Fig. 4A, potential binding sites were found between miR-508-3p and PTMA. In dual-luciferase reporter experiments, co-transfection of WT-PTMA with miR-508-3p mimic reduced luciferase activity, while co-transfection of MUT-PTMA with miR-508-3p mimic had no effect on luciferase activity (Fig. 4B). RIP experiments showed that miR-508-3p and PTMA were enriched in the Ago2 group (Fig. 4C). After hypoxia induction, the expression of PTMA was significantly decreased in SMMC-7721 cells (Fig. 4D). Furthermore, knockdown of circDNAH14 reduced PTMA expression, whereas knockdown of miR-508-3p had the opposite effect (Fig. 4E). These results suggest that miR-508-3p targets PTMA.

### Down-regulating PTMA promotes the effect of hypoxia on HCC cellS

**Figure 5:**
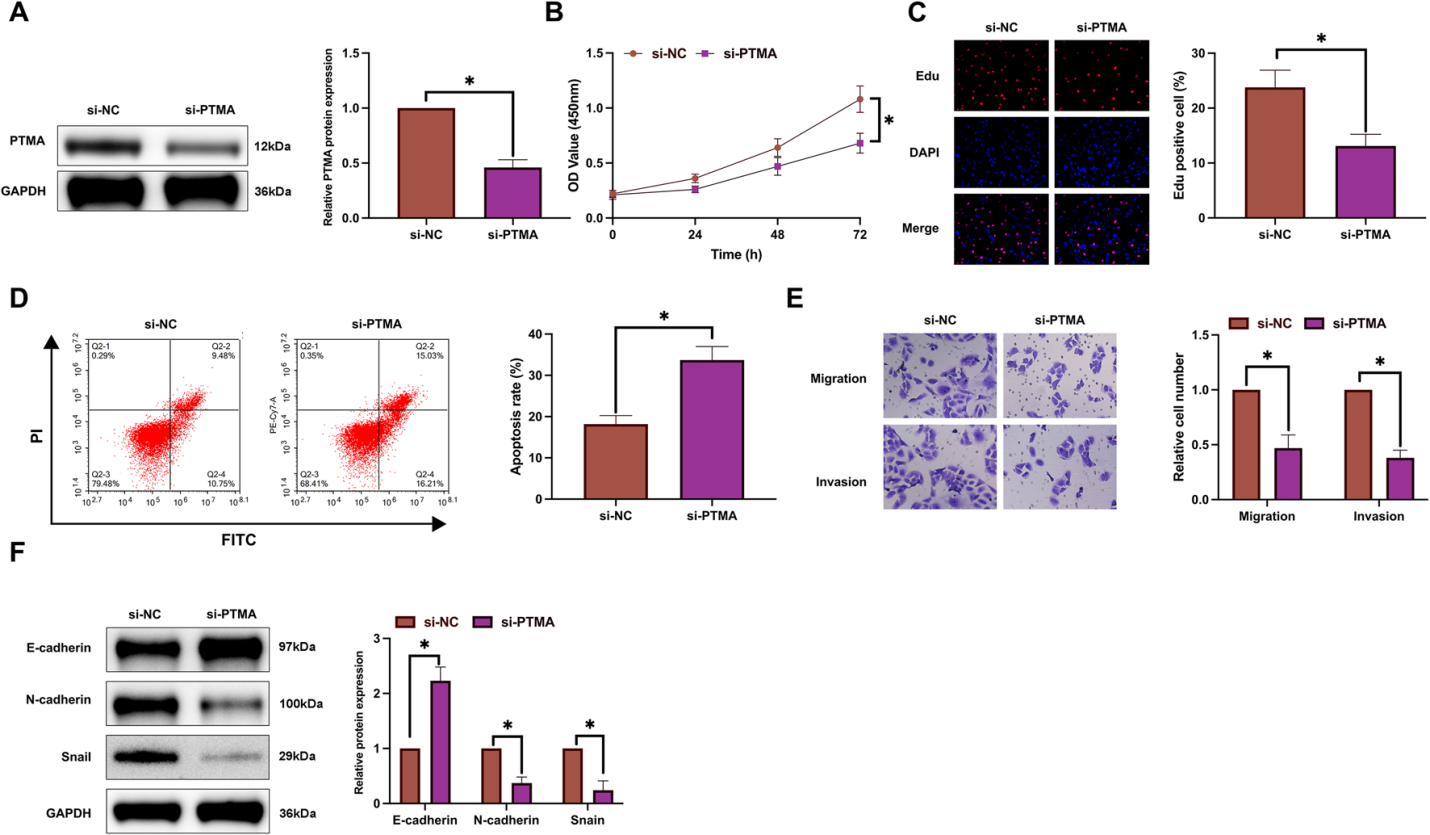
Depression of PTMA enhances the effect of hypoxia on HCC cells. PTMA in SMMC-7721 cells was silenced during hypoxia induction. (**A**) Western blot evaluation of PTMA expression; (**B**) CCK-8 detection of cell proliferation; (**C**) EdU analysis detection of cell DNA replication; (**D**) Flow cytometry detection of cell apoptosis; (**E**) Transwell detection of cell invasion and migration abilities; (**F**) Western blot detection of E-cadherin, N-cadherin, and Snail in cells; Data are presented as mean ± SD (N = 3). *P < 0.05.

PTMA was knocked down by convoluted siRNA targeting PTMA in hypoxic SMMC-7721 cells (Fig. 5A). Functional experiments showed that after knockdown of PTMA, the proliferation rate of hypoxic SMMC-7721 cells decreased (Fig. 5B, C), apoptosis rate increased (Fig. 5D), the number of invasive and migrating cells decreased (Fig. 5E), N-cadherin and Snail expression decreased while E-cadherin expression increased (Fig. 5F). These results suggest that knockdown of PTMA promotes the inhibitory effect of hypoxia on HCC.

### CircDNAH14 affects the inhibitory effect of hypoxia on HCC by regulating miR-508-3p/PTMA axis

**Figure 6:**
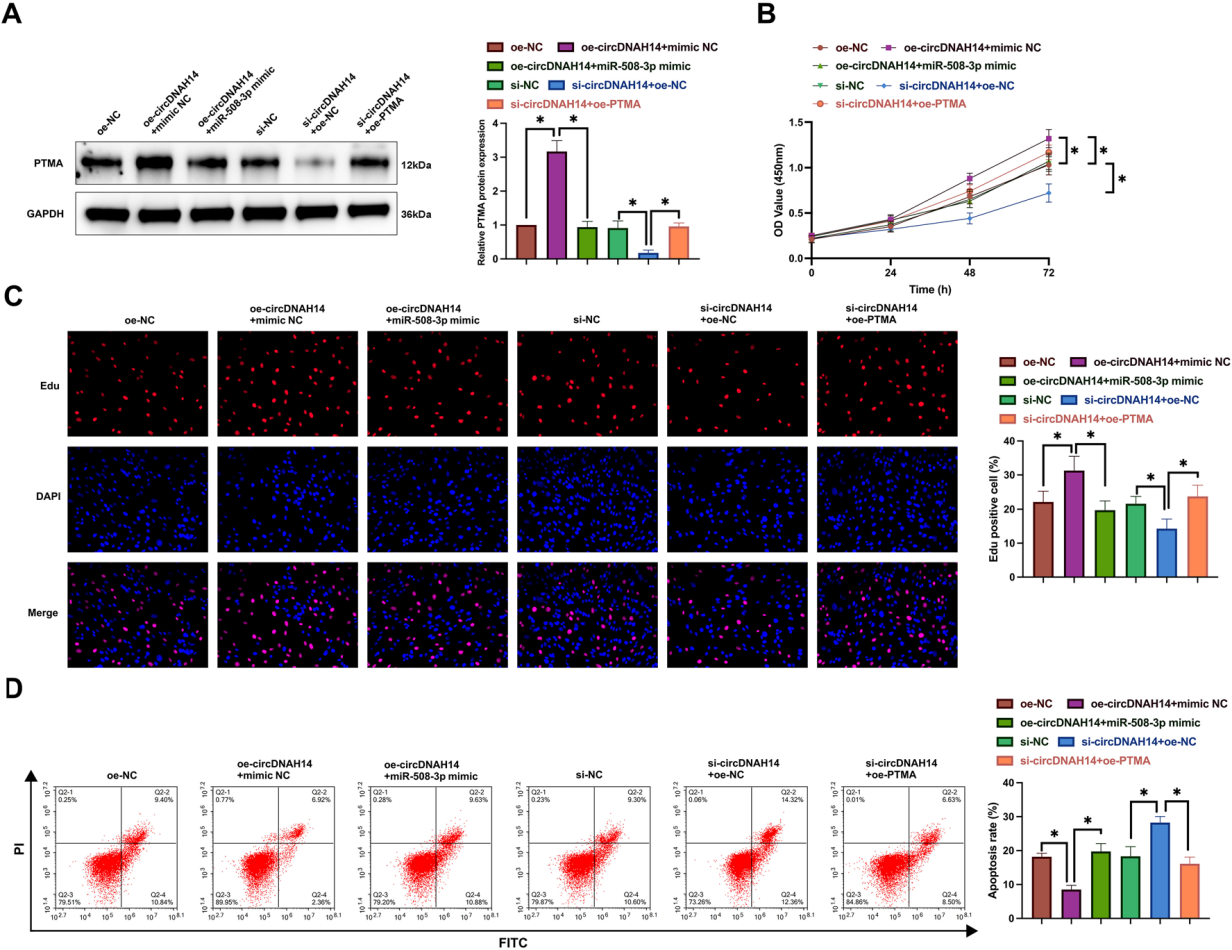
CircDNAH14 affects the inhibitory effect of hypoxia on HCC cells by regulating miR-508-3p/PTMA axis. oe-circDNAH14 and miR-508-3p, or si-circDNAH14 and oe-PTMA were co-transfected in hypoxic SMMC-7721 cells. (**A**) Western blot detection of PTMA; (**B**) CCK-8 detection of cell proliferation; (**C**) EdU analysis detection of cell DNA replication; (**D**) Flow cytometry detection of cell apoptosis; Data are presented as mean ± SD (N = 3). *P < 0.05.

Next, the role of the circDNAH14/miR-508-3p/PTMA axis was explored in hypoxic HCC cells. oe-circDNAH14, miR-508-3p mimic, si-circDNAH14, and oe-PTMA were co-transfected into hypoxic SMMC-7721 cells. Western blot showed that the promoting effect of oe-circDNAH14 on PTMA expression was reversed by miR-508-3p mimic, and the inhibitory effect of si-circDNAH14 on PTMA expression was reversed by oe-PTMA (Fig. 6A). Functional experiments showed that oe-circDNAH14 promoted cell proliferation and the number of EdU-positive cells (Fig. 6B,C), decreased apoptosis rate (Fig. 6D), increased invasion and migration ability (Supplementary Fig. 3B), promoted N-cadherin and Snail and inhibited E-cadherin expression (Supplementary Fig. 3C), but these effects were reversed by miR-508-3p mimic. Furthermore, si-circDNAH14 exhibited opposite effects to oe-circDNAH14, which suppressed the growth of SMMC-7721 cells, while oe-PTMA reversed the effects of si-circDNAH14 (Fig. 6B–D, Supplementary Fig. 3B,C). These results suggest that the circDNAH14/miR-508-3p/PTMA axis exerts a crucial role in hypoxia-suppressed HCC cell growth.

### Depression of circDNAH14 restrains HCC cell growth with hypoxia exposure in vivo

**Figure 7:**
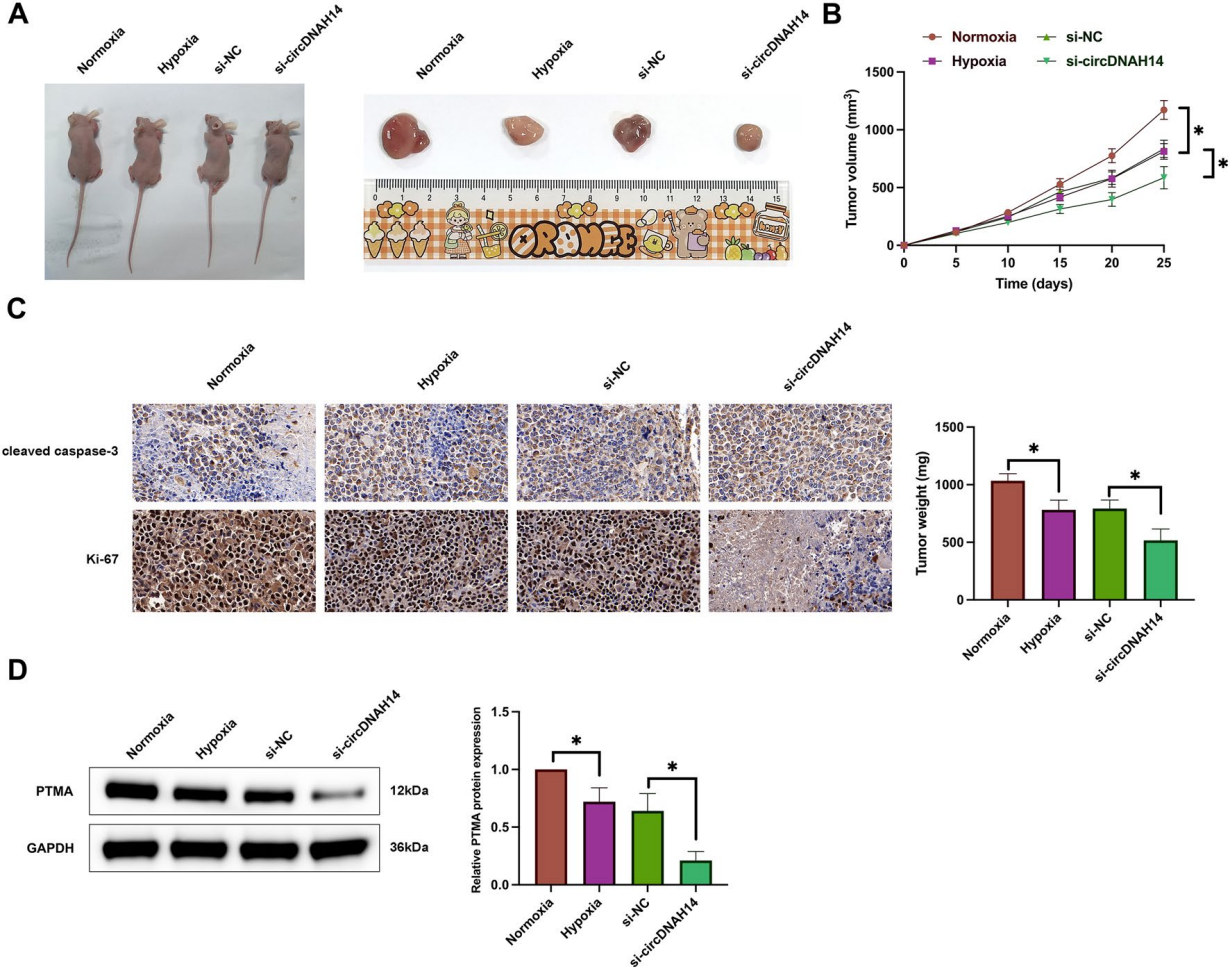
Refraining circDNAH14 restrains HCC cell growth with hypoxia exposure in vivo. (**A**) Representative images of tumors; (**B**) Tumor volume; (**C**) Immunohistochemistry detection of Ki-67 and cleaved caspase-3 in the tumor; Data are presented as mean ± SD (N = 3). *P < 0.05; (**D**) Western blot detection of PTMA in the tumor.

Subsequently, in vivo experiments were conducted to examine the effect of knockdown of circDNAH14 on the growth of hypoxia-exposed HCC cells. Hypoxia exposure suppressed tumor volume and tumor size, and knockdown of circDNAH14 aggravated this effect (Fig. 7A,B). Immunohistochemistry showed that hypoxia exposure resulted in an increase in cleaved caspase-3-positive cells and a decrease in Ki-67-positive cells in tumors, while knockdown of circDNAH14 promoted these effects of hypoxia exposure (Fig. 7C). Western blot showed that PTMA expression was reduced in tumors after hypoxia exposure, while knockdown of circDNAH14 further suppressed HMBG1 expression (Fig. 7D). These results clarify that depression of circDNAH14 restrains HCC cell growth with hypoxia exposure in vivo.

## Discussion

CircRNA is involved in sorts of biological processes in tumors via interacting with its miRNA response elements^23^. HCC is one of the most common cancers and is highly refractory to most systemic therapies^24^. Accumulating evidence suggests that circRNAs play a key role in HCC^25^, but something remains to be explored regarding the impacts of circRNAs on HCC in the treatment of TACE. In this study, circDNAH14 affects the biological behaviors of HCC cells under hypoxic conditions by regulating the miR-508-3p/PTMA axis.

Multiple circRNAs have been confirmed to be differentially expressed in HCC. For example, a recent study found that 236 circRNAs were differentially expressed in HCC compared to matched normal tissues, with 108 up-regulated and 128 down-regulated^26^. In addition, circRNA regulates the malignant phenotype of liver cancer cells through multiple pathways. For example, circSMARCA5 acts as a sponge for miR-17-3p and miR-181b-5p to promote the expression of tissue inhibitor of metalloproteinases 3, while down-regulation of circSMARCA5 promotes the proliferation and metastasis of HCC^27^. circBACH1 has been found to be significantly upregulated in HCC tissues and interacts with HuR (a widely studied RBP) to promote HuR translocation and promote its accumulation in the cytoplasm, thereby downregulating p27 expression^28^. Qin et al*.*^29^ report that 390 and 489 circRNAs were differentially expressed during TACE, suggesting that circRNAs can be used as indicators to predict the efficacy of TACE, such as circ-004213. Liu et al*.*^30^ reported that lncRNA myocardial infarction-associated transcript expression is enhanced in tumors of HCC patients receiving TACE and hypoxia-stimulated HCC cells. The theoretical basis of TACE in the treatment of HCC is based on the difference in blood supply between liver cancer and normal liver tissue. The blood supply of HCC mainly comes from the hepatic artery, while that of normal liver tissue is from the portal vein^31^*.*

circRNA frequently performs as miRNAs’ sponges in HCC^32^. The research discovered that circDNAH14 had binding sites with miR-508-3p. MiR-508-3p is a gene situated at the Xq27.3 locus of the human X chromosome and has been discovered to be involved in gastric^33^, colorectal^34^, and cervical cancers^35^. The research originally found that hypoxia elevated miR-508-3p expression, and knockdown of miR-508-3p attenuated the inhibitory effect of hypoxia on HCC cells, while overexpression of miR-508-3p reversed the effect of overexpression of circDNAH14 and enhanced the inhibitory effect of hypoxia on HCC cells.

PTMA was miR-508-3p’s downstream target gene in the study. PTMA, a nuclear oncoprotein transcription factor, is essential for cell cycle progression and proliferation. PTMA is elevated in HCC and is applied as a prognostic marker^36^. Lin et al*.*^37^ suggested that PTMA expression is enhanced in HCC and can protect HCC cells from cell death stimulated by sorafenib. Additionally, PTMA has also been confirmed to be implicated in the cisplatin (DDP) resistance of HCC cells^38^. The research also observed that PTMA was overexpressed in HCC, whereas hypoxia significantly repressed PTMA expression. Moreover, depression of PTMA promoted the inhibitory effect of hypoxia on HCC cells, while overexpression of PTMA reversed the effect of down-regulation of circDNAH14 and attenuated the inhibitory effect of hypoxia on HCC cells.

It is pertinent to note that our study is preliminary and primarily relies on a cobalt chloride-induced hypoxia model. This reliance constrains the direct clinical applicability of our findings. Notably, the clinical deployment of intracellular molecular biomarkers is hindered by the challenges associated with serial biopsies of patient tumors, a practice often contraindicated in HCC treatment. While our research proposes the potential significance of circDNAH14 in TACE treatment response, current evidence does not sufficiently demonstrate its distinct regulatory mechanisms in HCC patients or its efficacy as a prognostic biomarker. Future investigations are warranted to validate our findings in clinical samples and to explore the potential synergy of circDNAH14 with other treatment modalities, such as pharmacological sensitization. Although TACE procedures may induce hypoxia in tumor tissues, our study design did not capture the direct impact of chemotherapeutic agents on circDNAH14 expression. Future research should incorporate this aspect to more comprehensively understand the molecular mechanisms in TACE treatment.

## Conclusion

Taken together, the results of this study suggest that circDNAH14 expression is reduced in hypoxic HCC cells. circDNAH14 attenuated the inhibitory effect of hypoxia on HCC cells by competitively adsorbing miR-508-3p and mediating PTMA expression. The circDNAH14/miR-508-3p/PTMA axis may serve as a potential molecular target to enhance the efficacy of TACE in the future, which will help improve the survival prognosis of HCC patients treated with TACE. The disadvantage is that this study did not analyze the clinical samples of HCC patients who received TACE treatment, so the clinical effect of the circDNAH14/miR-508-3p/PTMA axis needs to be explored in the follow-up research. In addition, it is necessary to perform receiver operating characteristic analysis to characterize the diagnostic effect of circDNAH14 on the therapeutic effect of TACE.

### Supplementary Information

Supplementary Figures.

Supplementary Figure 1.

Supplementary Figure 3.

Supplementary Figure 4.

Supplementary Figure 4.

Supplementary Figure 5.

Supplementary Figure 5.

Supplementary Figure 6.

Supplementary Figure 7.

Supplementary Figure 3.

Supplementary Information 10.

Supplementary Information 11.

## Data Availability

The data that support the findings of this study are available from corresponding author but restrictions apply to the availability of these data, which were used under license for the current study, and so are not publicly available. Data are however available from the authors upon reasonable request and with permission of corresponding author.
