# Supplementary material for: Circular RNA DNAH14 molecular mechanism in an experimental model of hepatocellular carcinoma treated with Cobalt chloride to mimic the hypoxia-like response of transcatheter arterial chemoembolization
Source: Sci Rep. 2024 Jan 23;14:1992. doi: 10.1038/s41598-024-52578-3 (PMC10805718; doi:10.1038/s41598-024-52578-3)
Supplement: Supplementary file 1 — Supplementary Figures. [file 41598_2024_52578_MOESM1_ESM.docx]

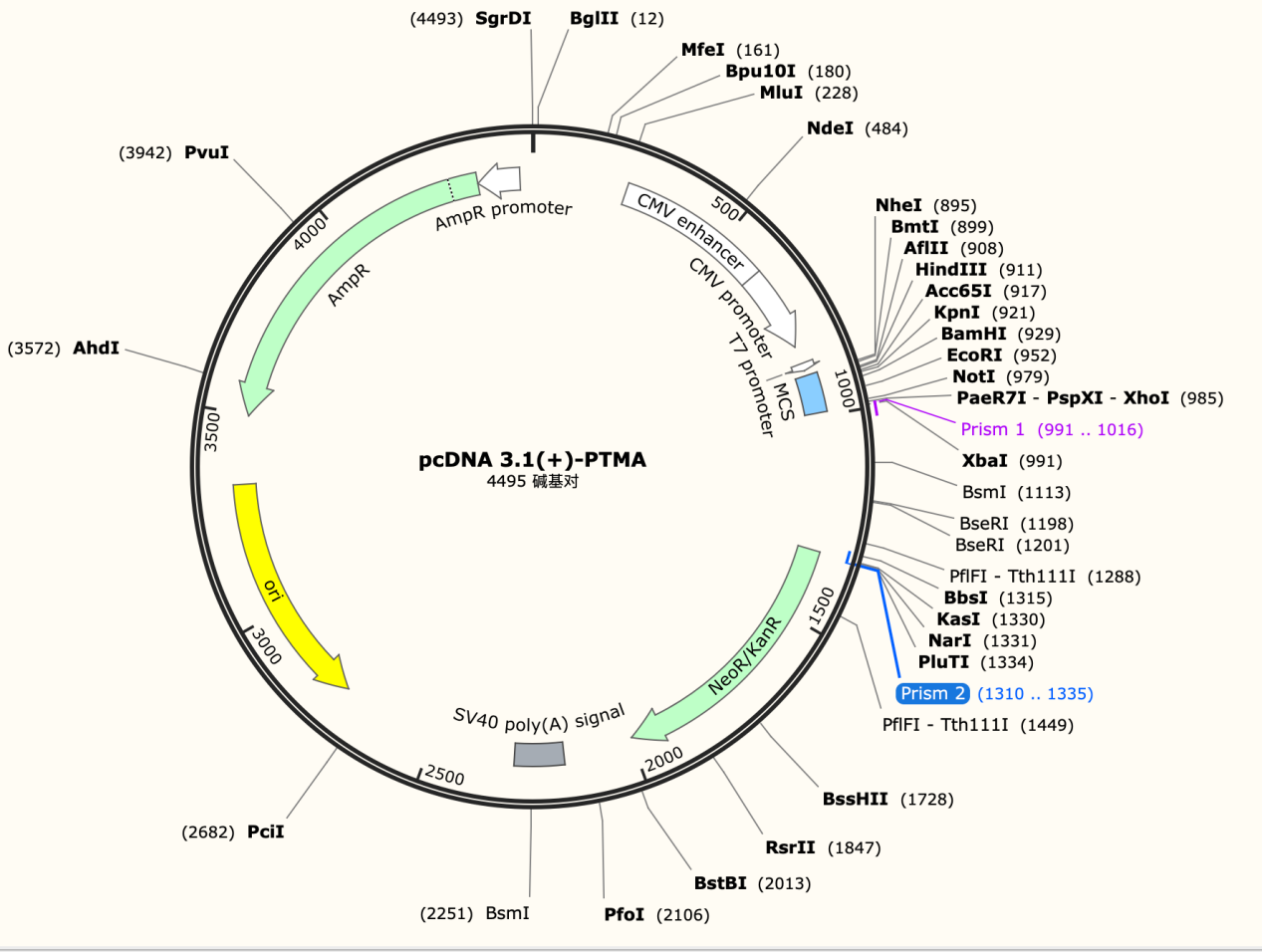


**Supplementary Fig. 1** **Plasmid information for pcDNA 3.1(+)-PTMA**


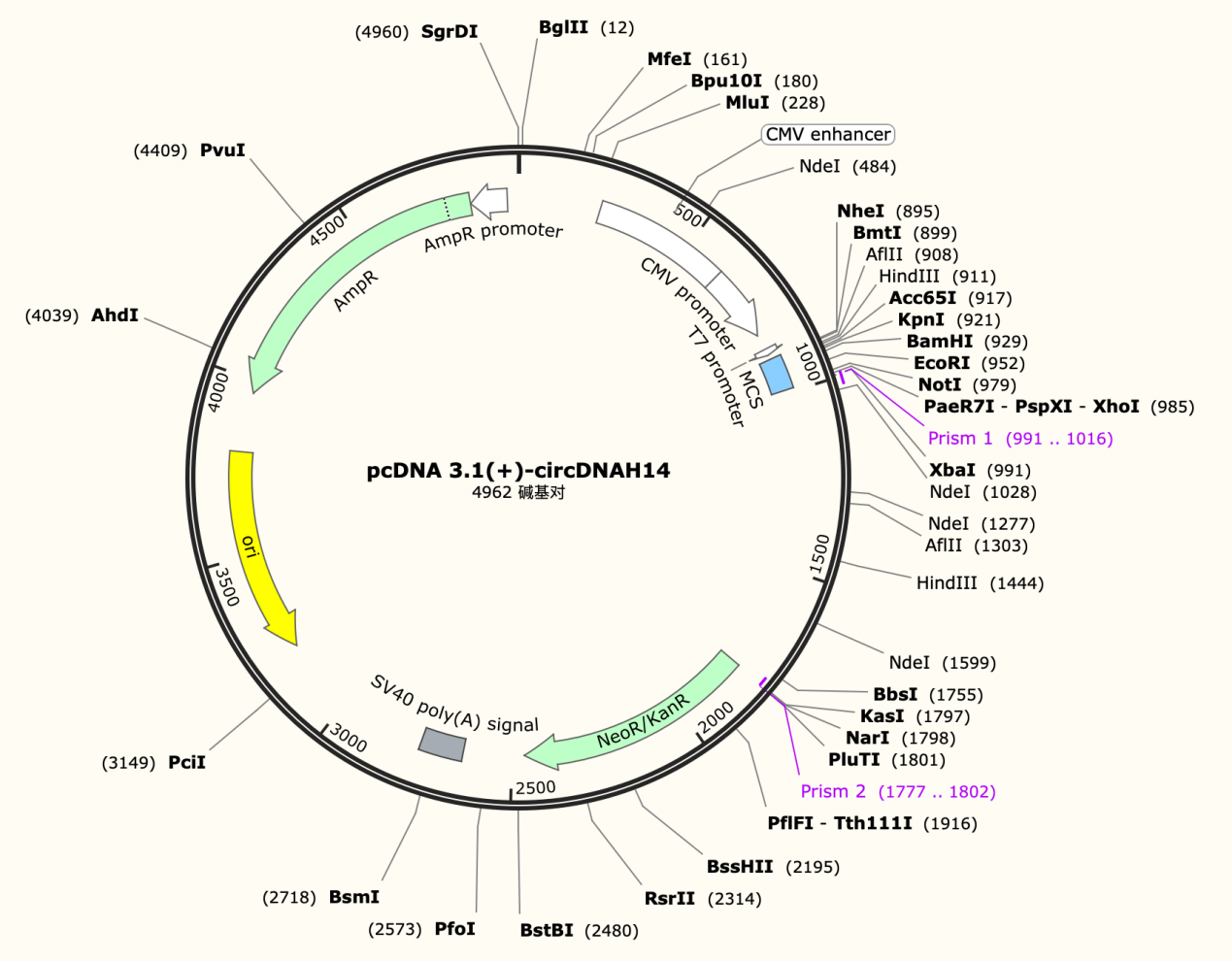


**Supplementary Fig. 2** **Plasmid information for pcDNA 3.1(+)-circDNAH14**


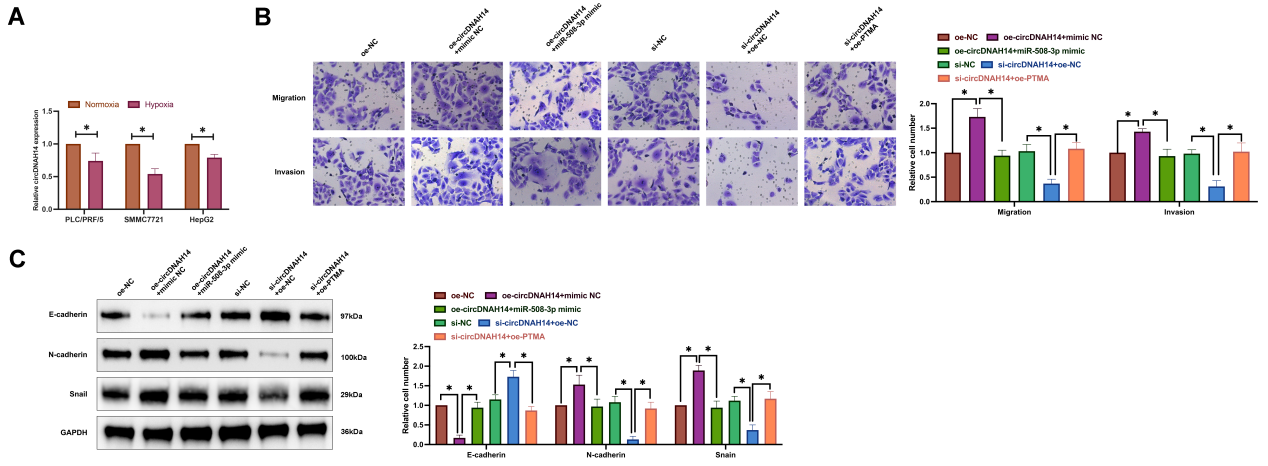


**Supplementary Fig. 3 CircDNAH14 affects the inhibitory effect of hypoxia on HCC by regulating miR-508-3p/PTMA axis**

A: circDNAH14 expression in HCC cell lines under hypoxia; B: Transwell detection of cell invasion and migration abilities; C: Western blot detection of E-cadherin, N-cadherin, and Snail in cells; Data are presented as mean ± SD (N = 3). * *P* < 0.05.
