# Supplementary material for: Circular RNA DNAH14 molecular mechanism in an experimental model of hepatocellular carcinoma treated with Cobalt chloride to mimic the hypoxia-like response of transcatheter arterial chemoembolization
Source: Sci Rep. 2024 Jan 23;14:1992. doi: 10.1038/s41598-024-52578-3 (PMC10805718; doi:10.1038/s41598-024-52578-3)
Supplement: Supplementary file 2 — Supplementary Figure 1. [file 41598_2024_52578_MOESM2_ESM.pdf]

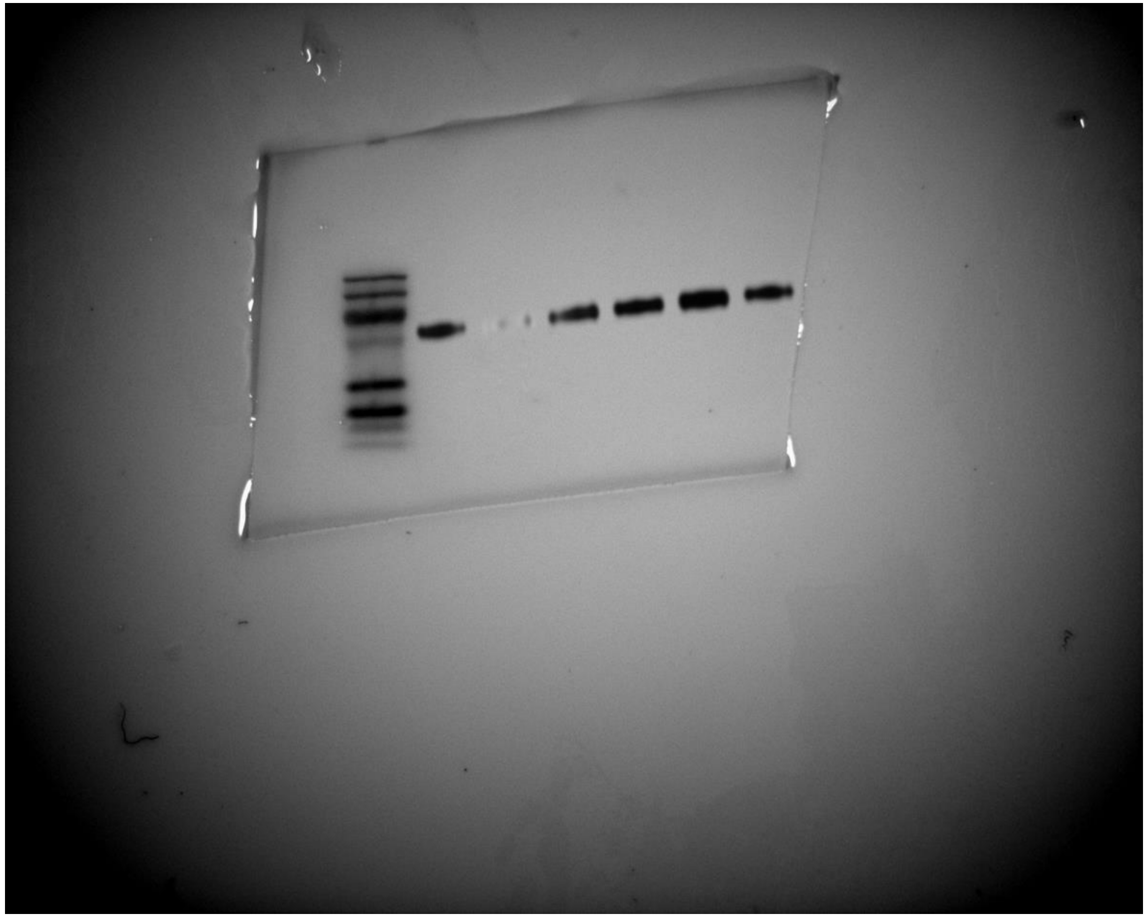

Supplementary Figure 3C E-cadherin

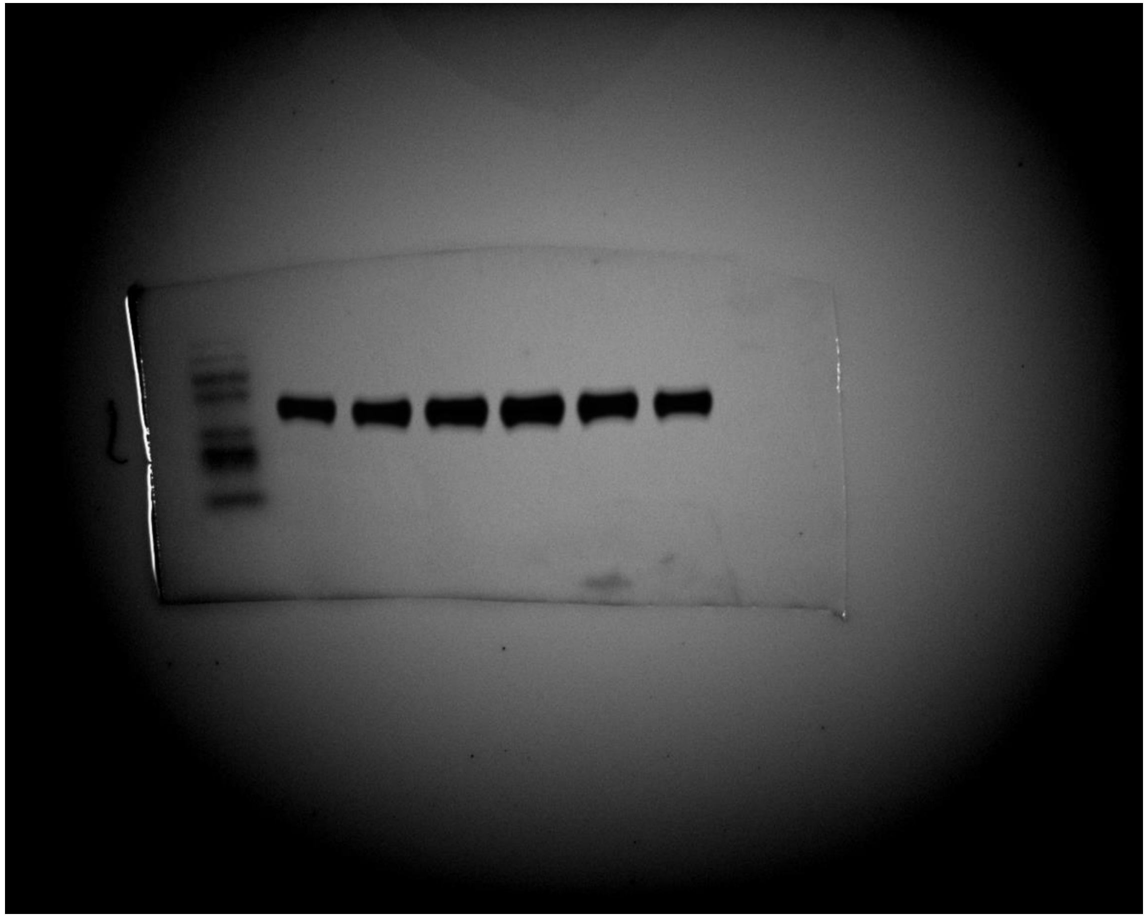

Supplementary Figure 3C GAPDH

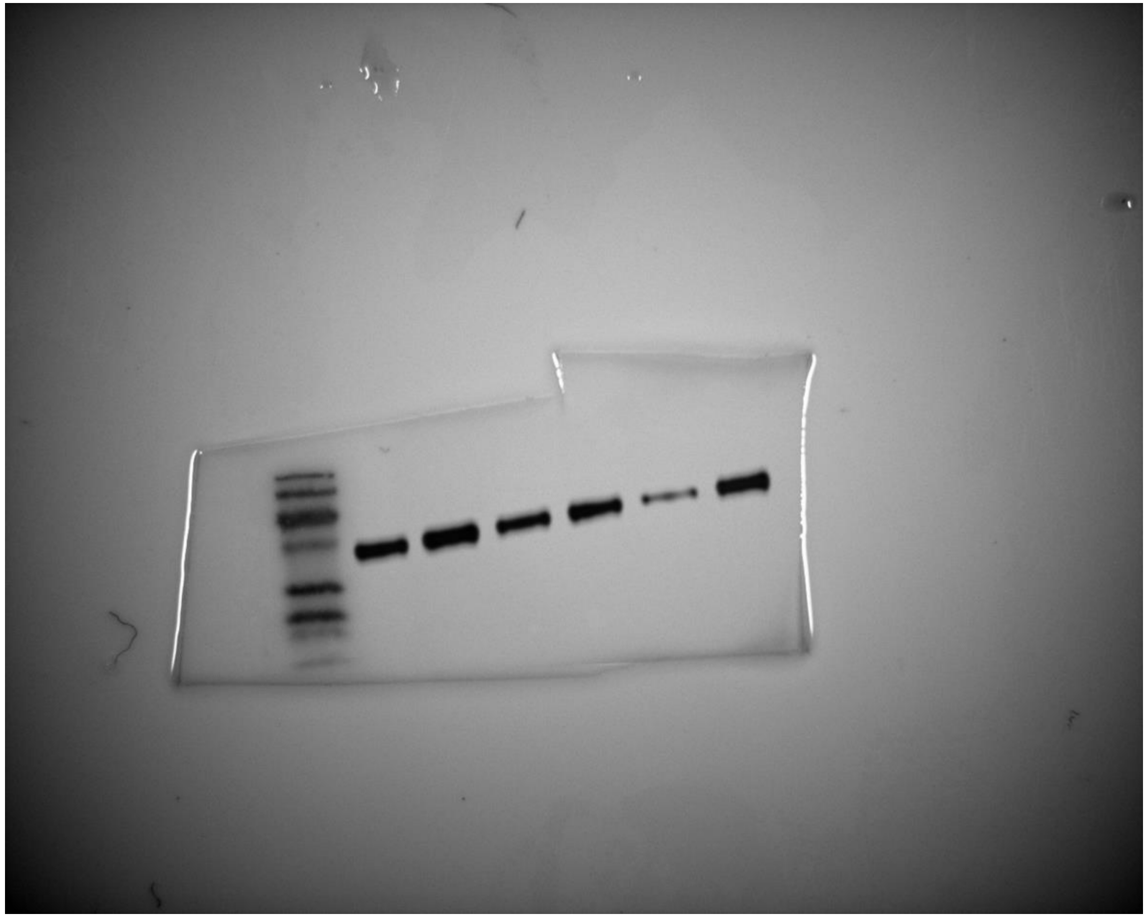

Supplementary Figure 3C N-cadherin

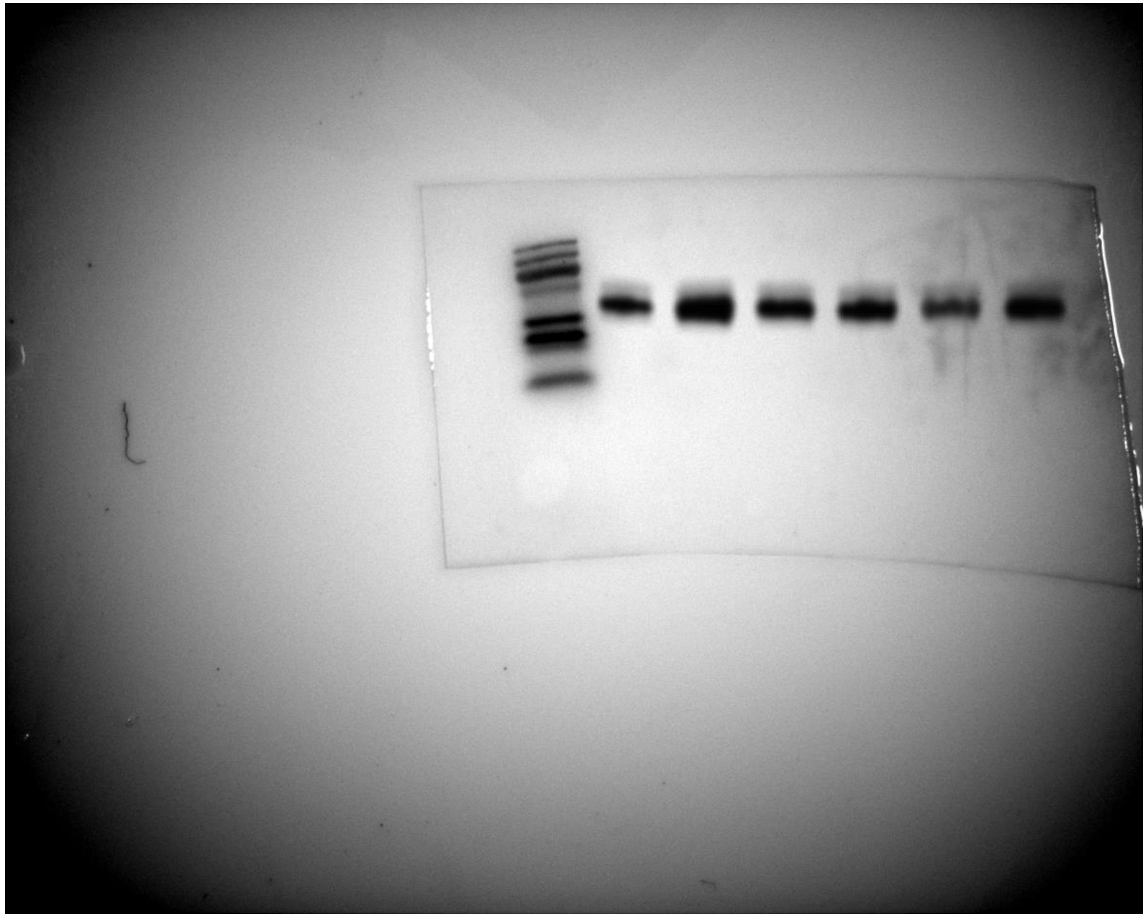

Supplementary Figure 3C Snail
