# Supplementary figures and images for: Circular RNA DNAH14 molecular mechanism in an experimental model of hepatocellular carcinoma treated with Cobalt chloride to mimic the hypoxia-like response of transcatheter arterial chemoembolization
Source: Sci Rep. 2024 Jan 23;14:1992. doi: 10.1038/s41598-024-52578-3 (PMC10805718; doi:10.1038/s41598-024-52578-3)

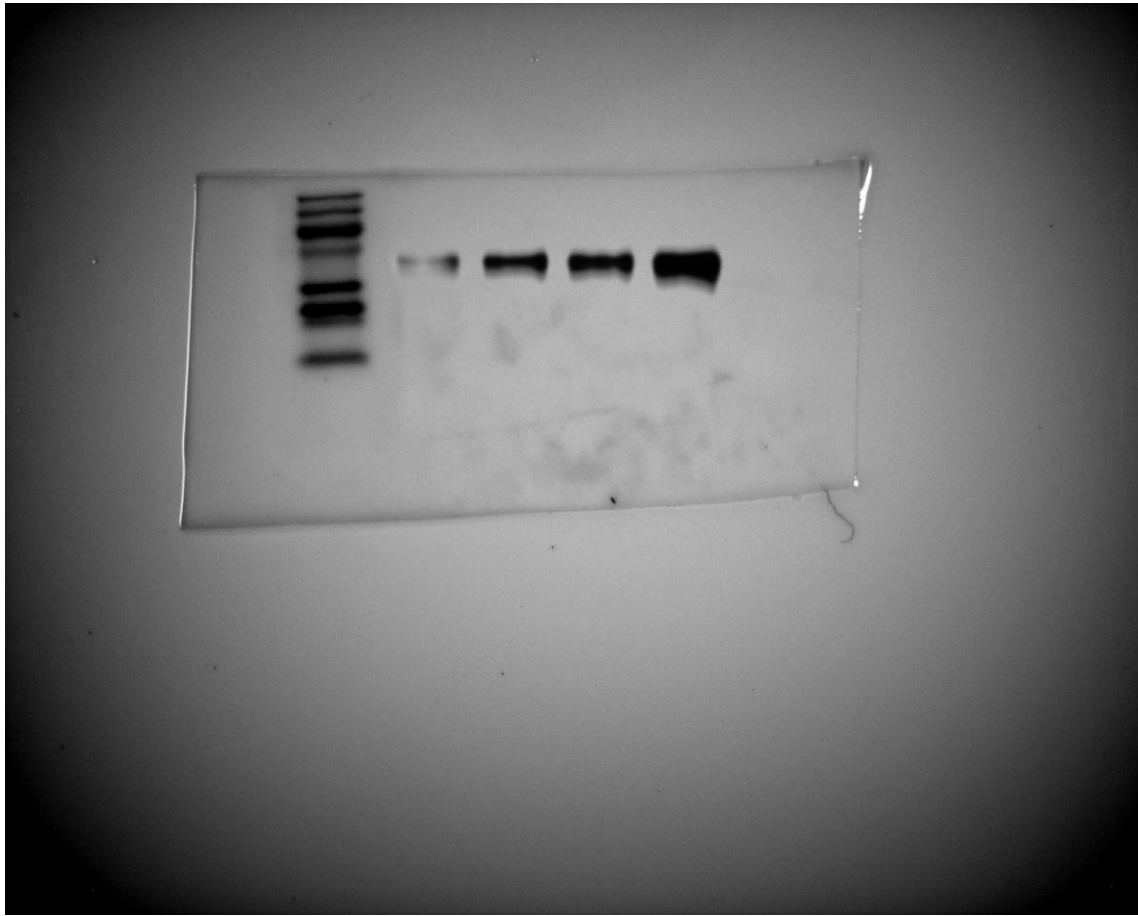

Figure 1G E-cadherin

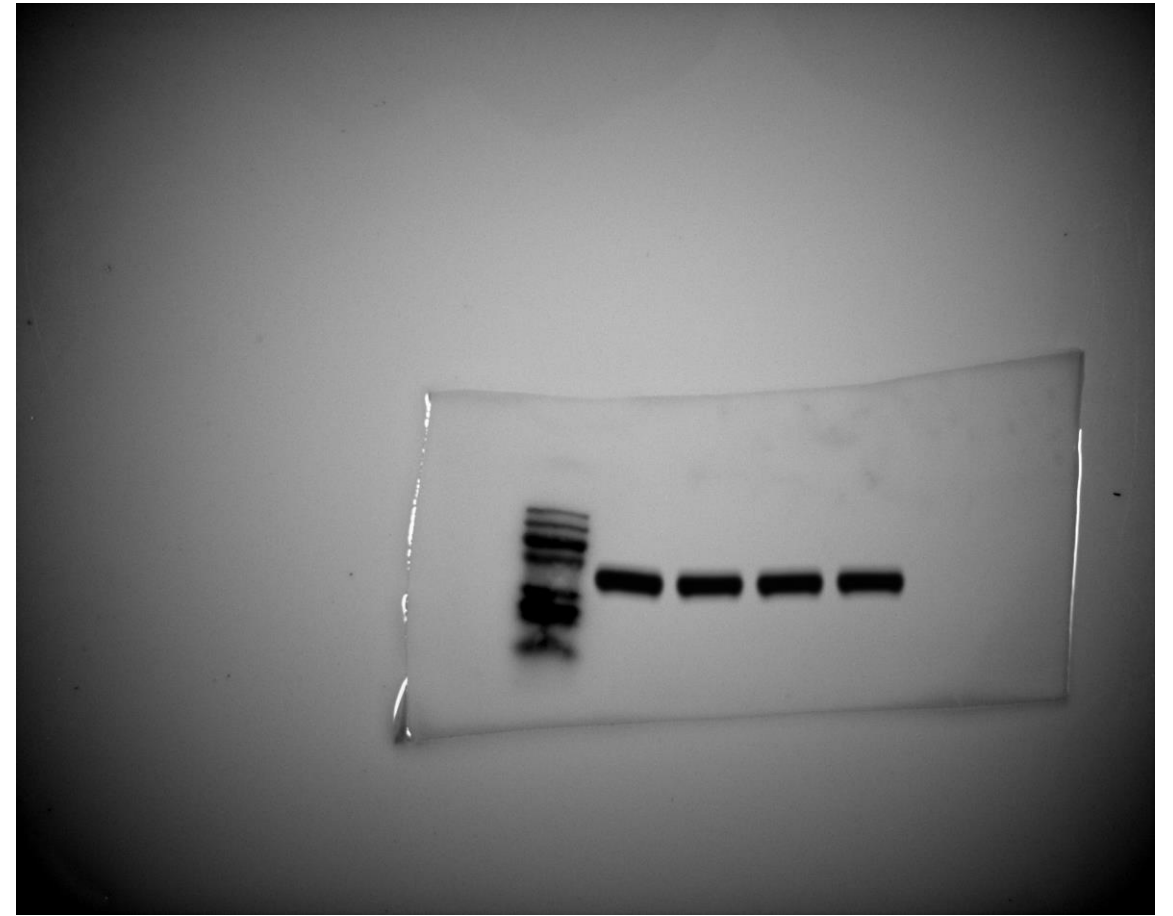

Figure 1G GAPDH

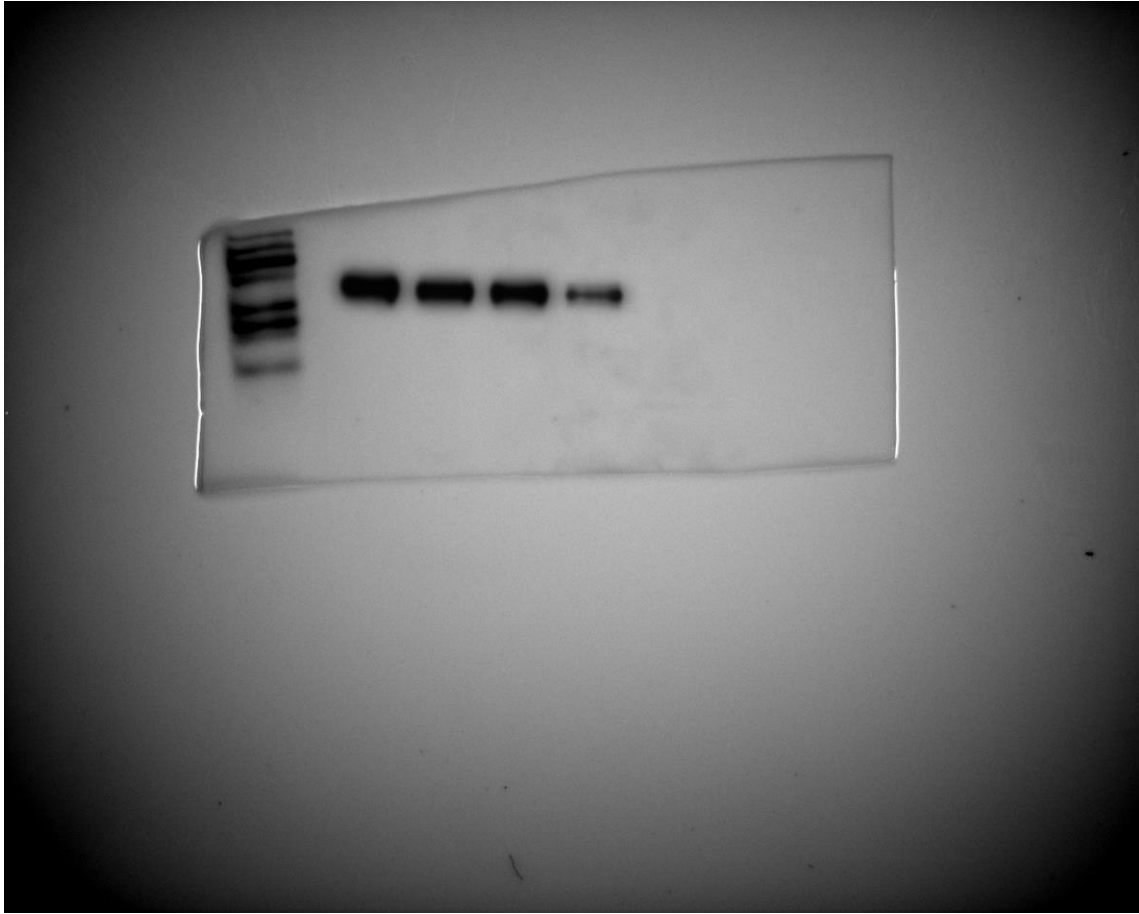

Figure 1G N-cadherin

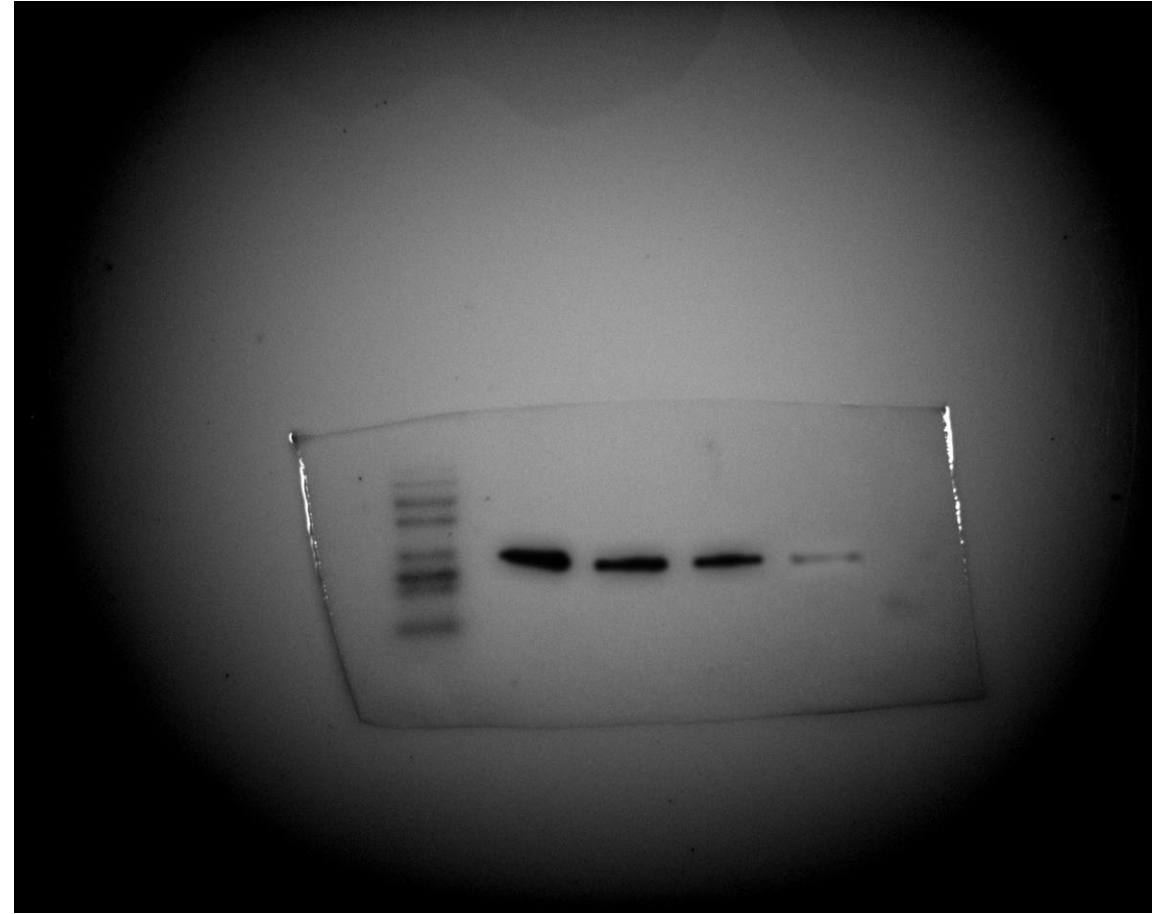

Figure 1G Snail

Supplement: Supplementary file 3 — Supplementary Figure 3. [file 41598_2024_52578_MOESM3_ESM.pdf]

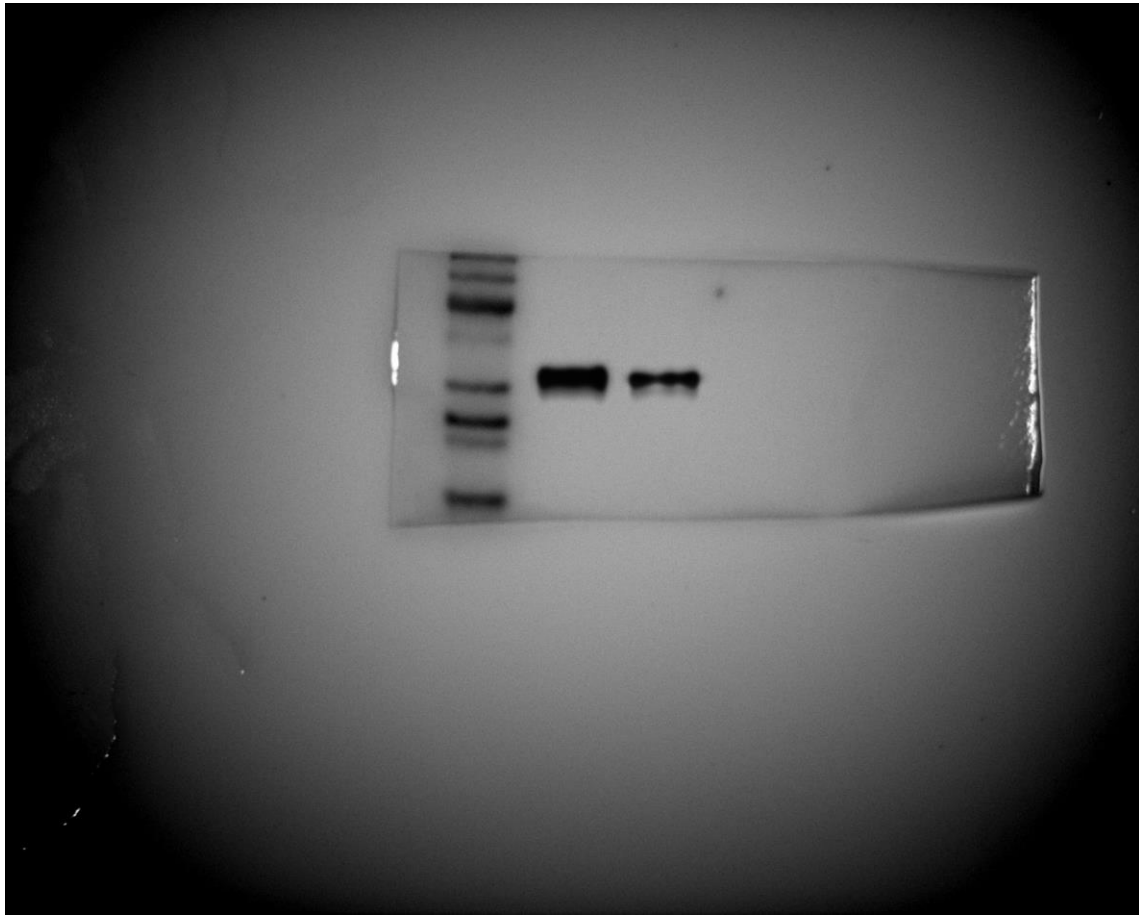

Figure 3F E-cadherin

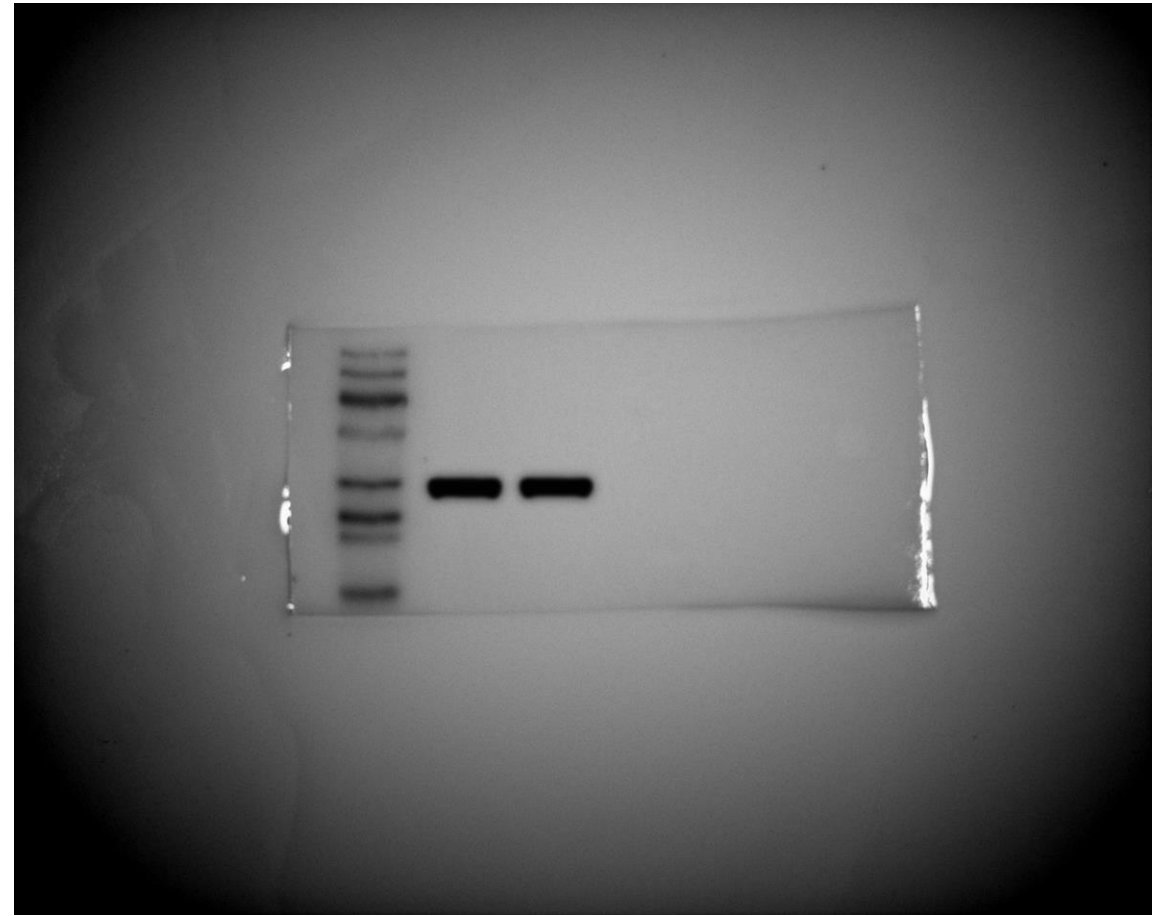

Figure 3F GAPDH

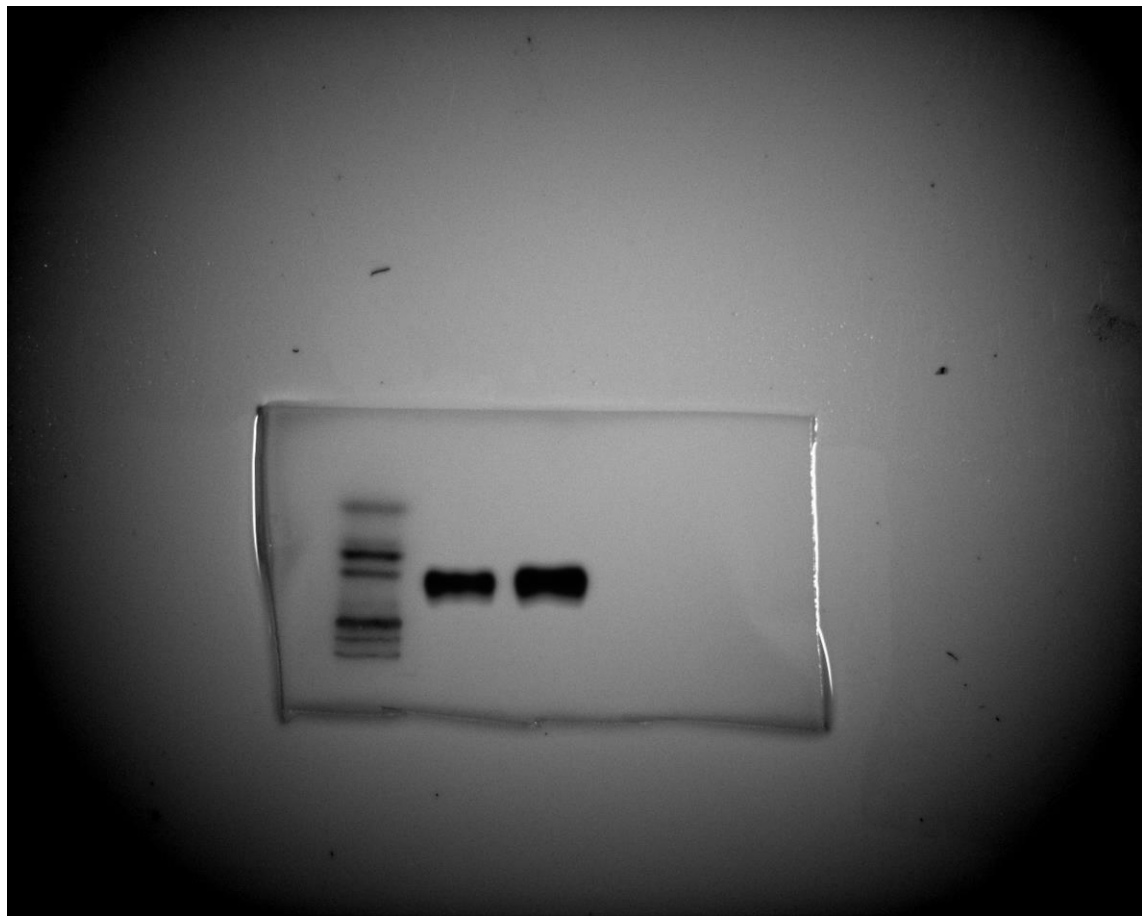

Figure 3F N-cadherin

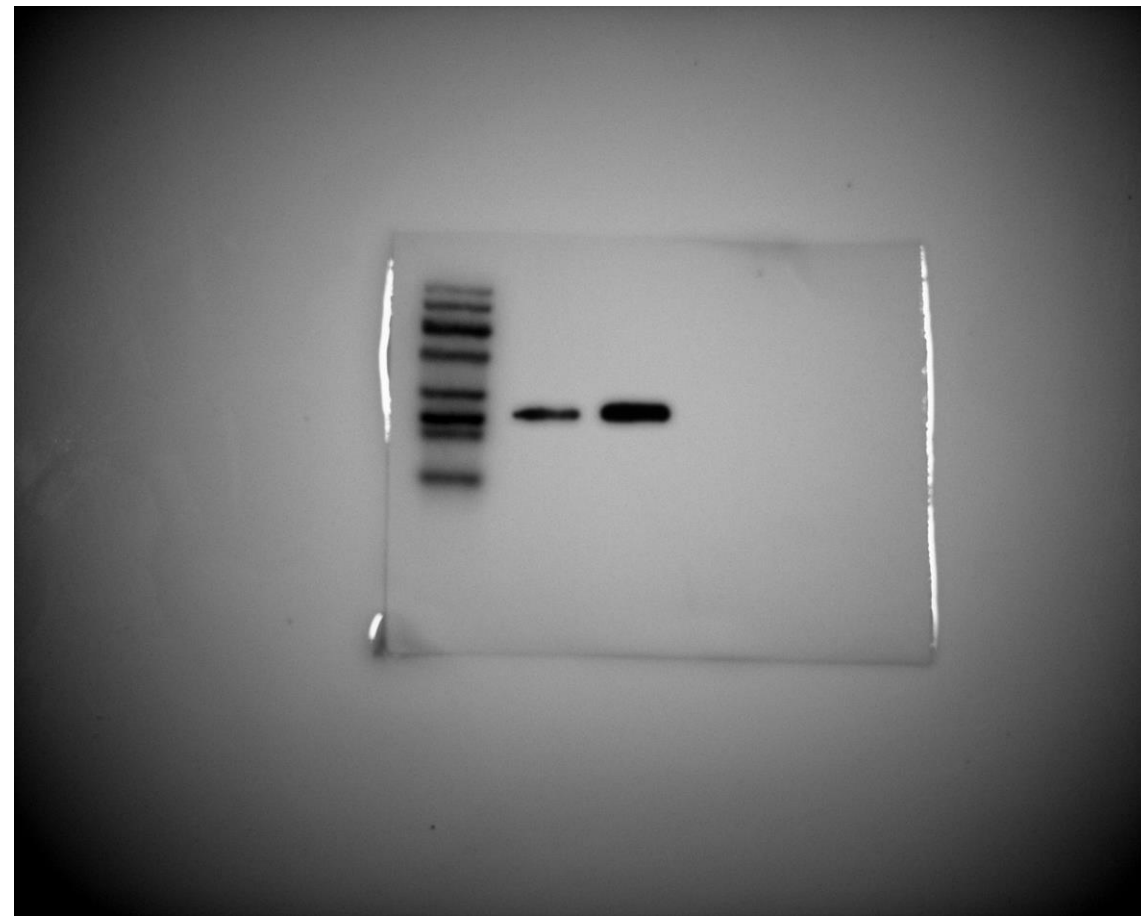

Figure 3F Snail

Supplement: Supplementary file 4 — Supplementary Figure 4. [file 41598_2024_52578_MOESM4_ESM.pdf]

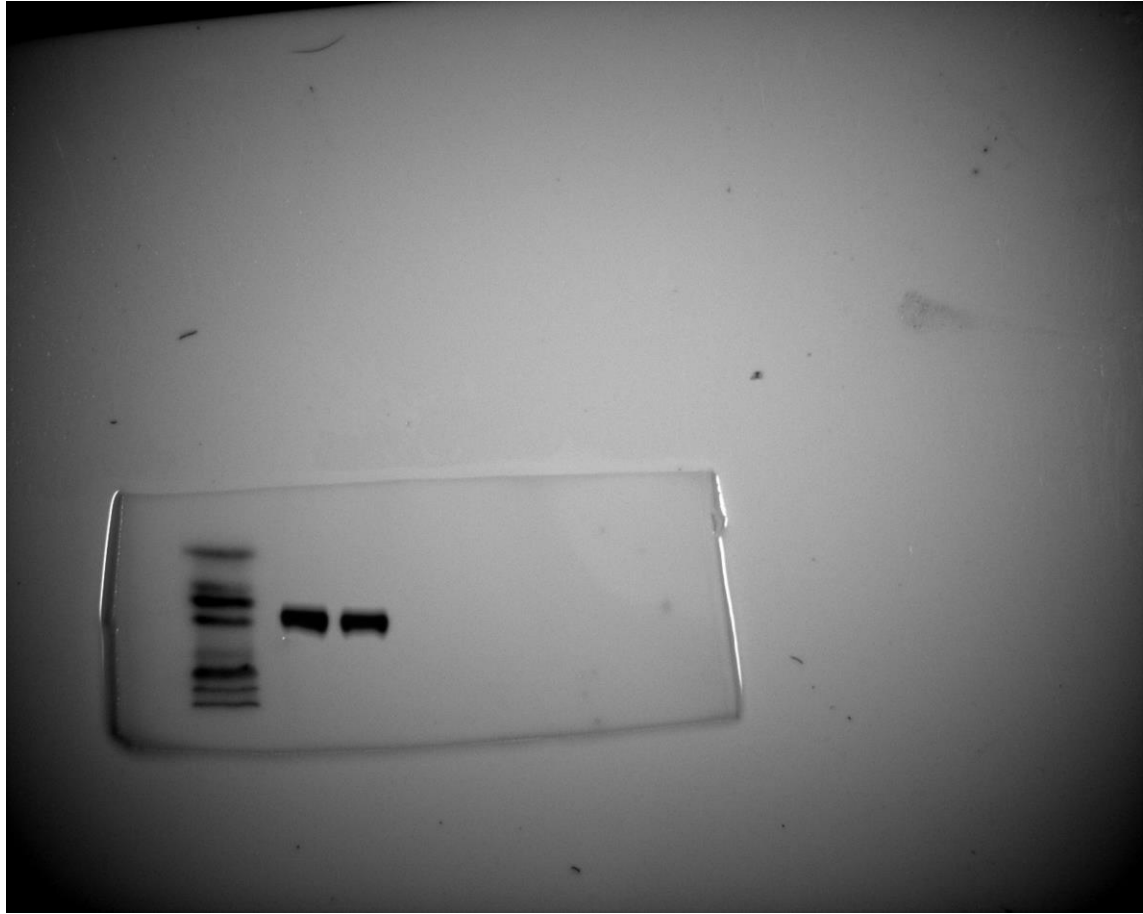

Figure 4D GAPDH

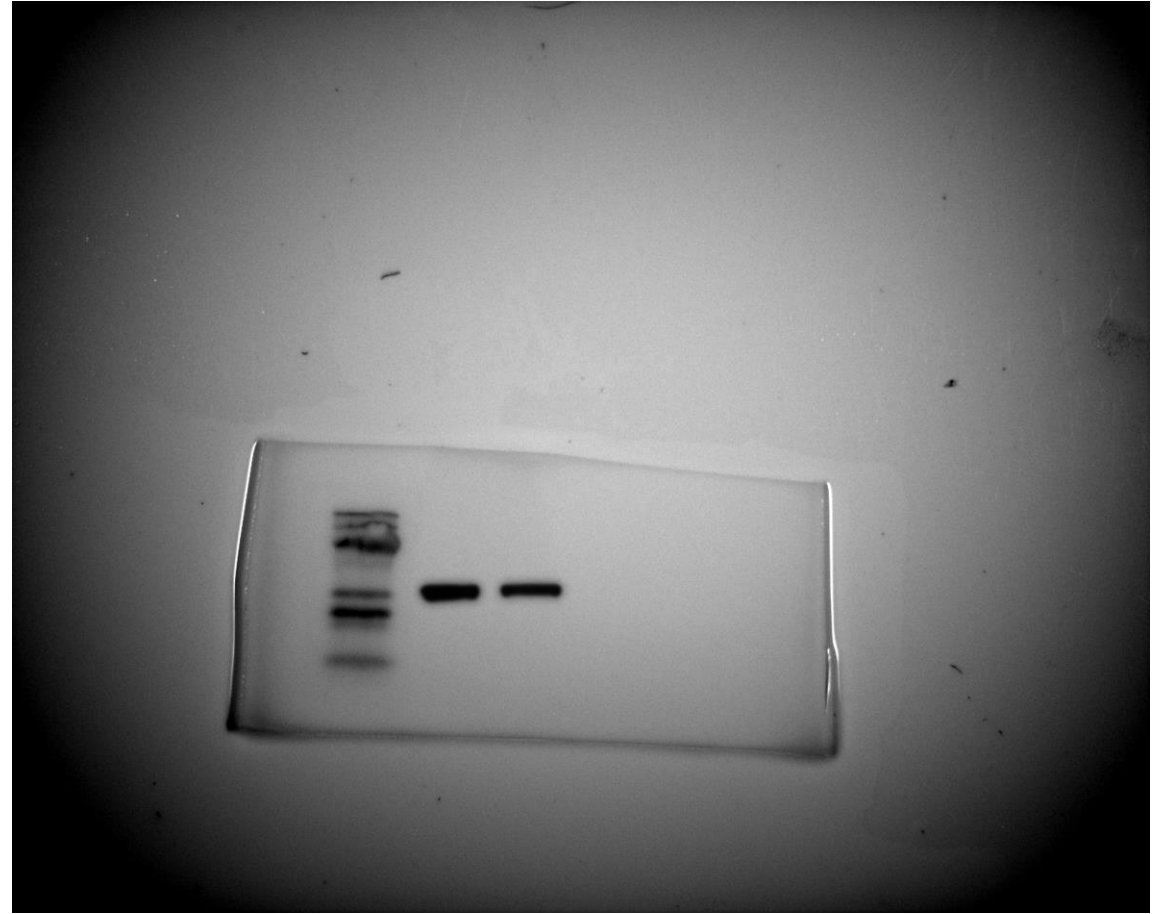

Figure 4D PTMA

Supplement: Supplementary file 5 — Supplementary Figure 4. [file 41598_2024_52578_MOESM5_ESM.pdf]

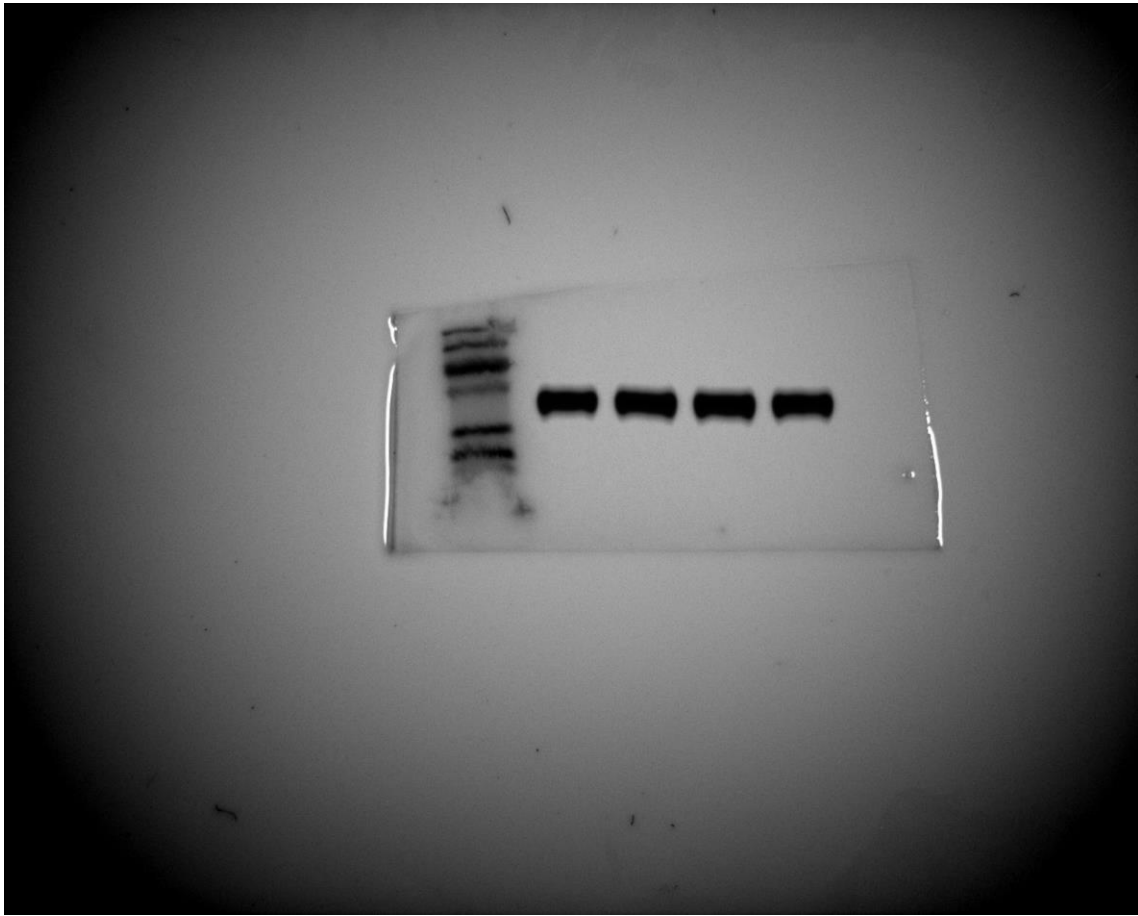

Figure 4E GAPDH

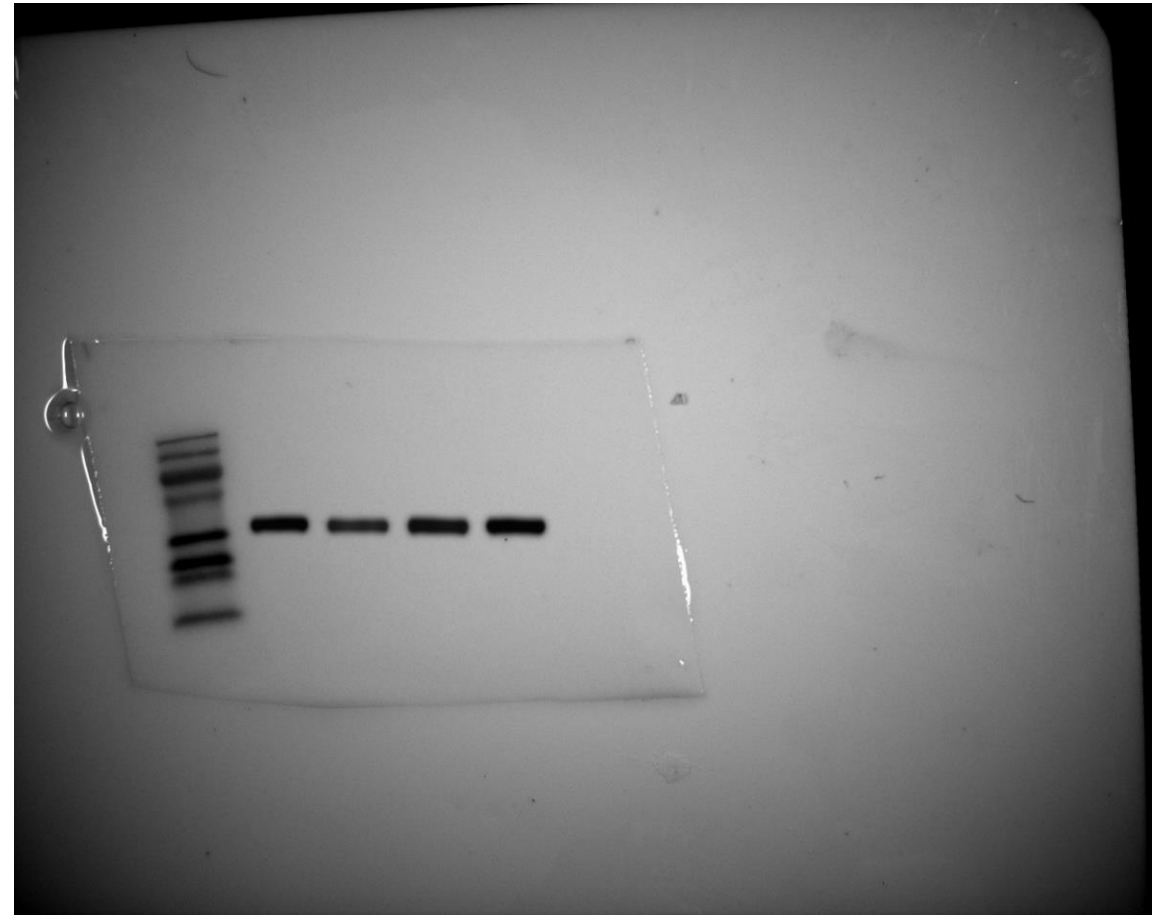

Figure 4E PTMA

Supplement: Supplementary file 6 — Supplementary Figure 5. [file 41598_2024_52578_MOESM6_ESM.pdf]

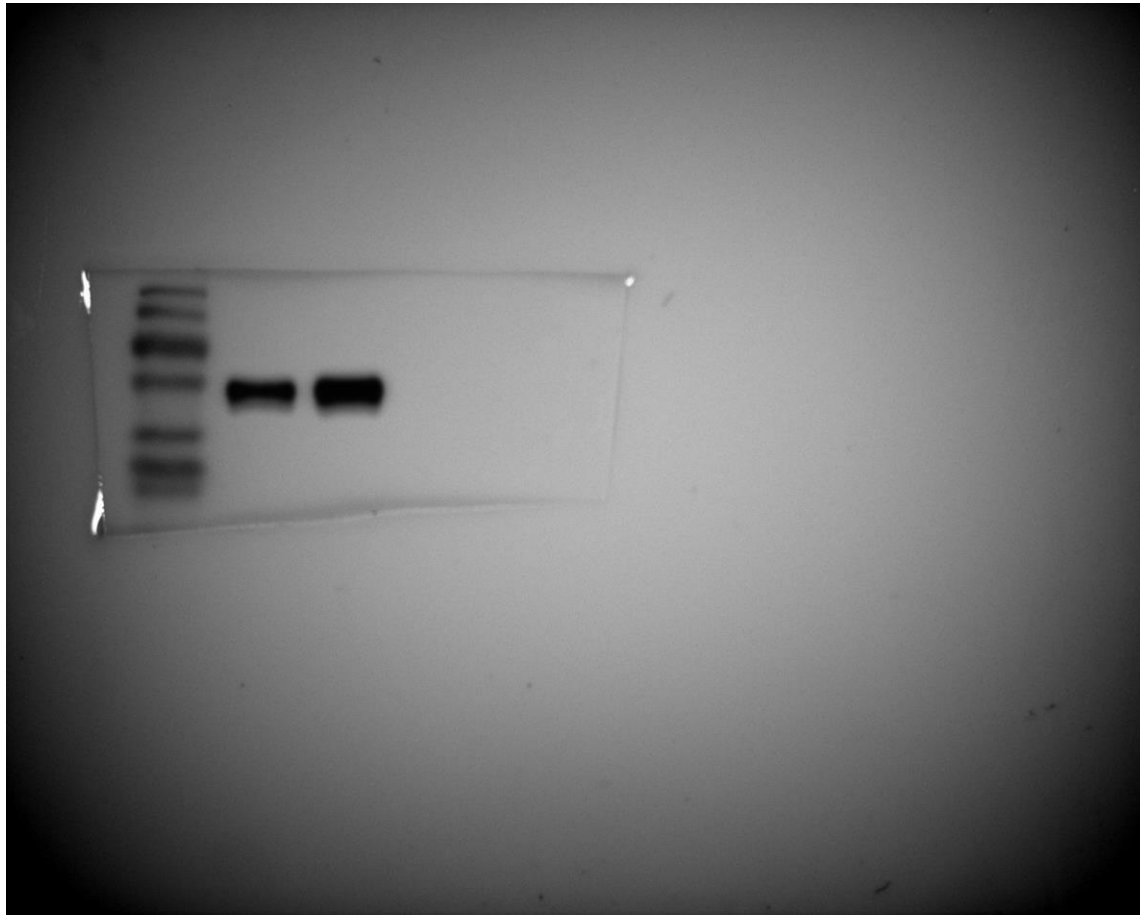

Figure 5F E-cadherin

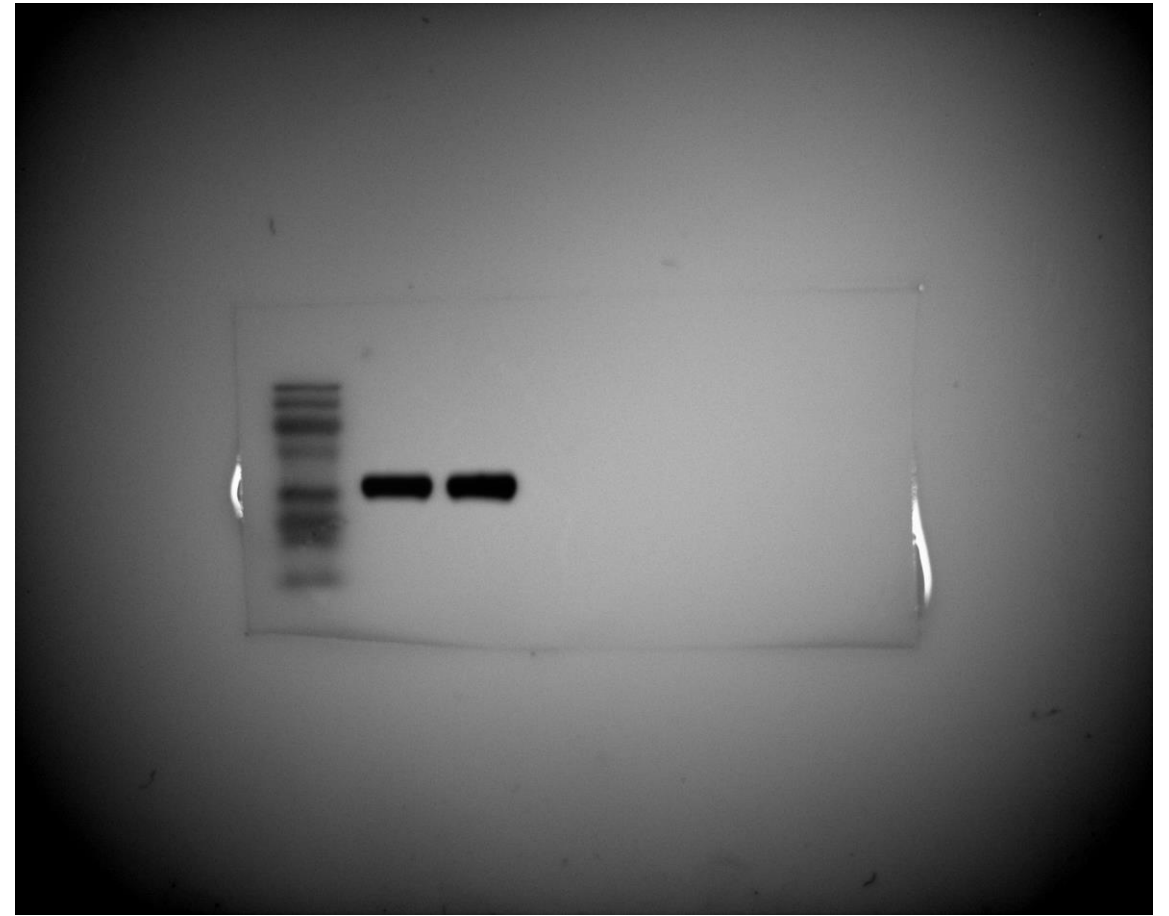

Figure 5F GAPDH

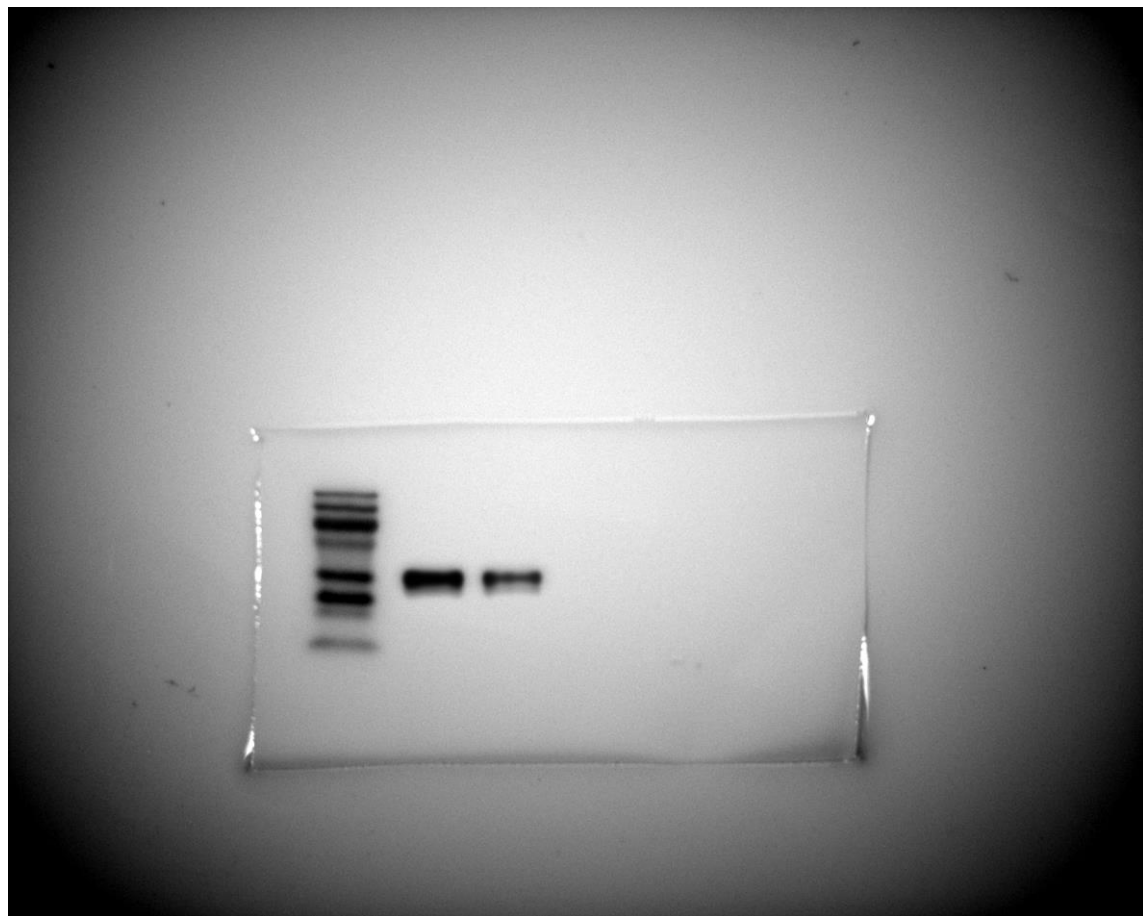

Figure 5F N-cadherin

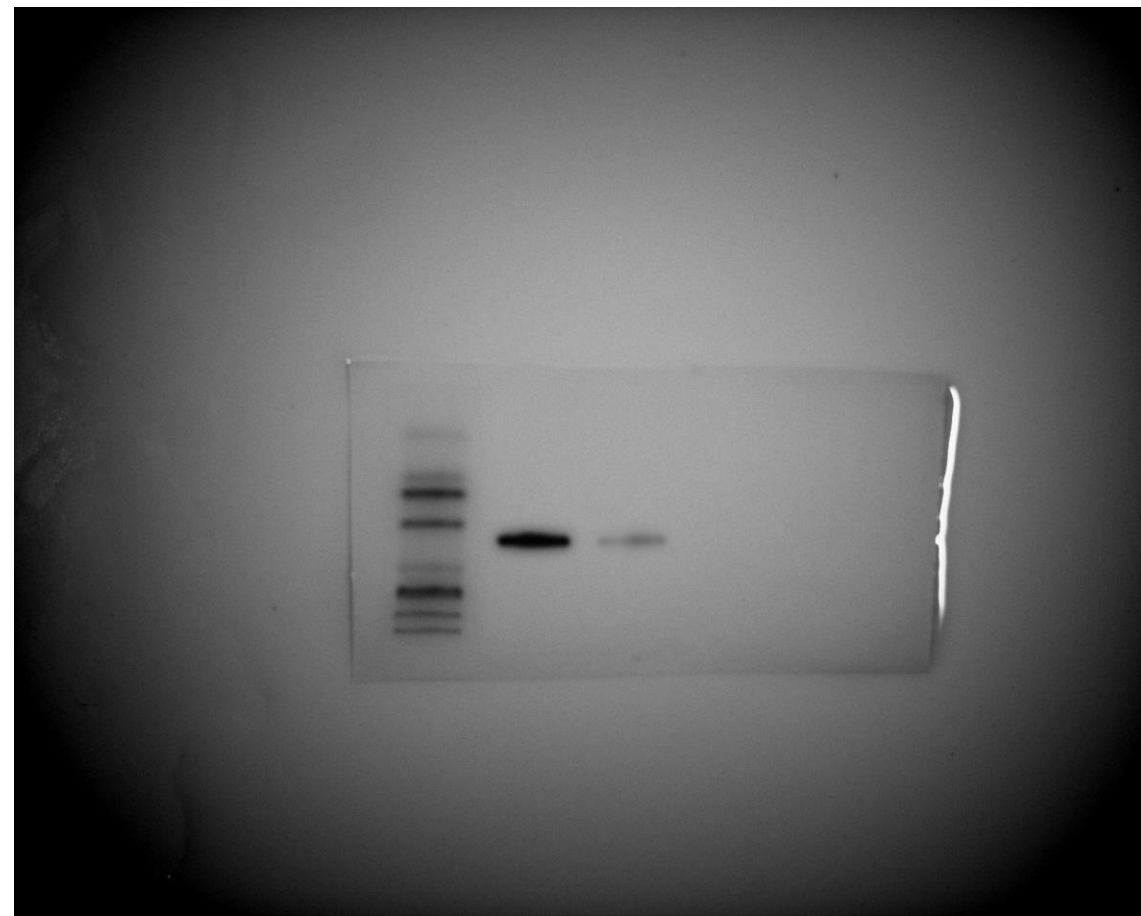

Figure 5F Snail

Supplement: Supplementary file 7 — Supplementary Figure 5. [file 41598_2024_52578_MOESM7_ESM.pdf]

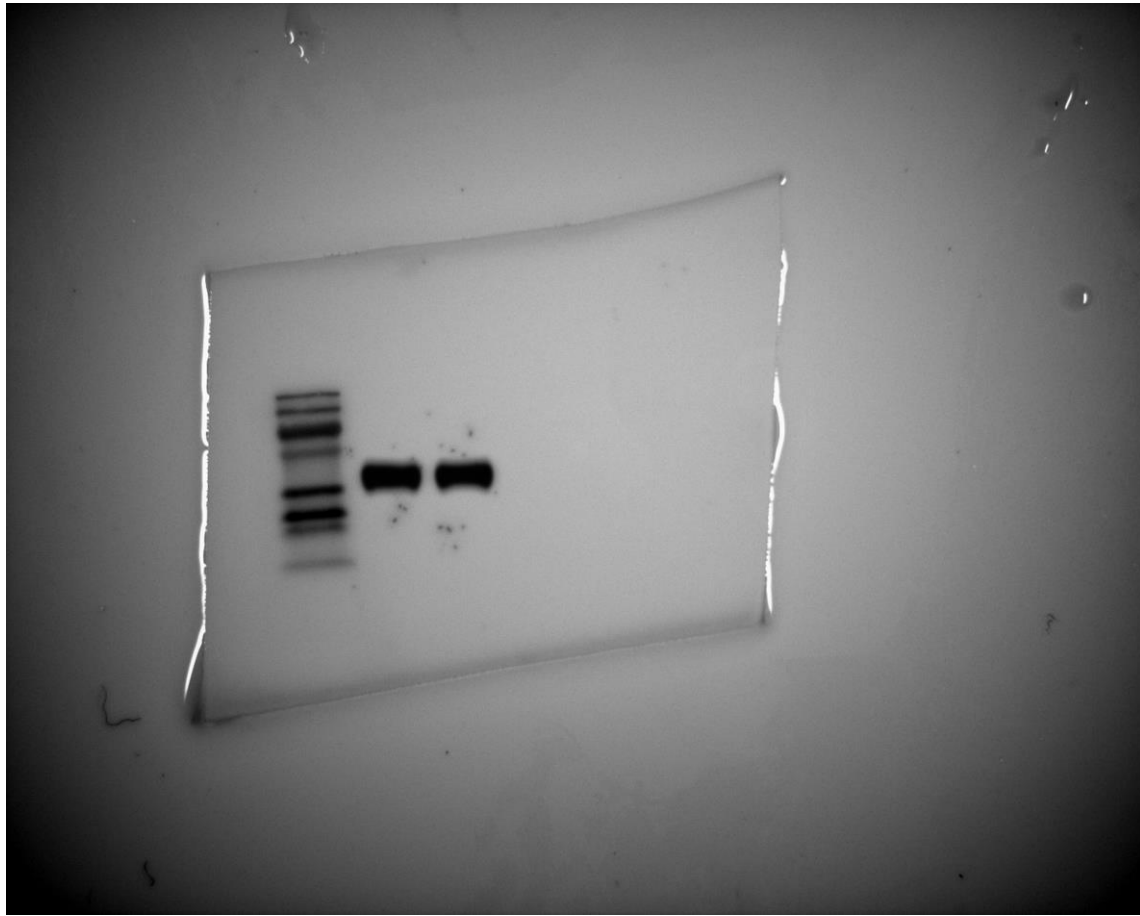

Figure 5A GAPDH

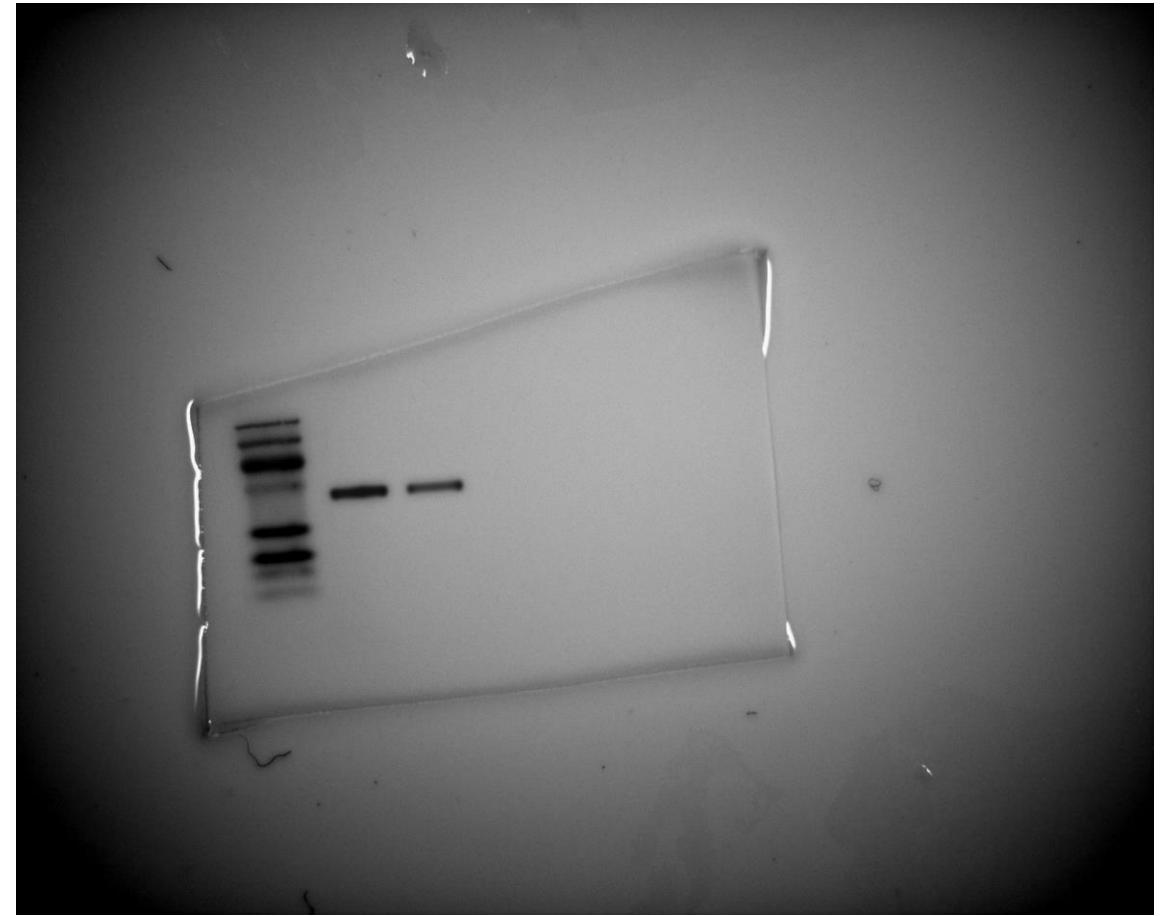

Figure 5A PTMA

Supplement: Supplementary file 8 — Supplementary Figure 6. [file 41598_2024_52578_MOESM8_ESM.pdf]

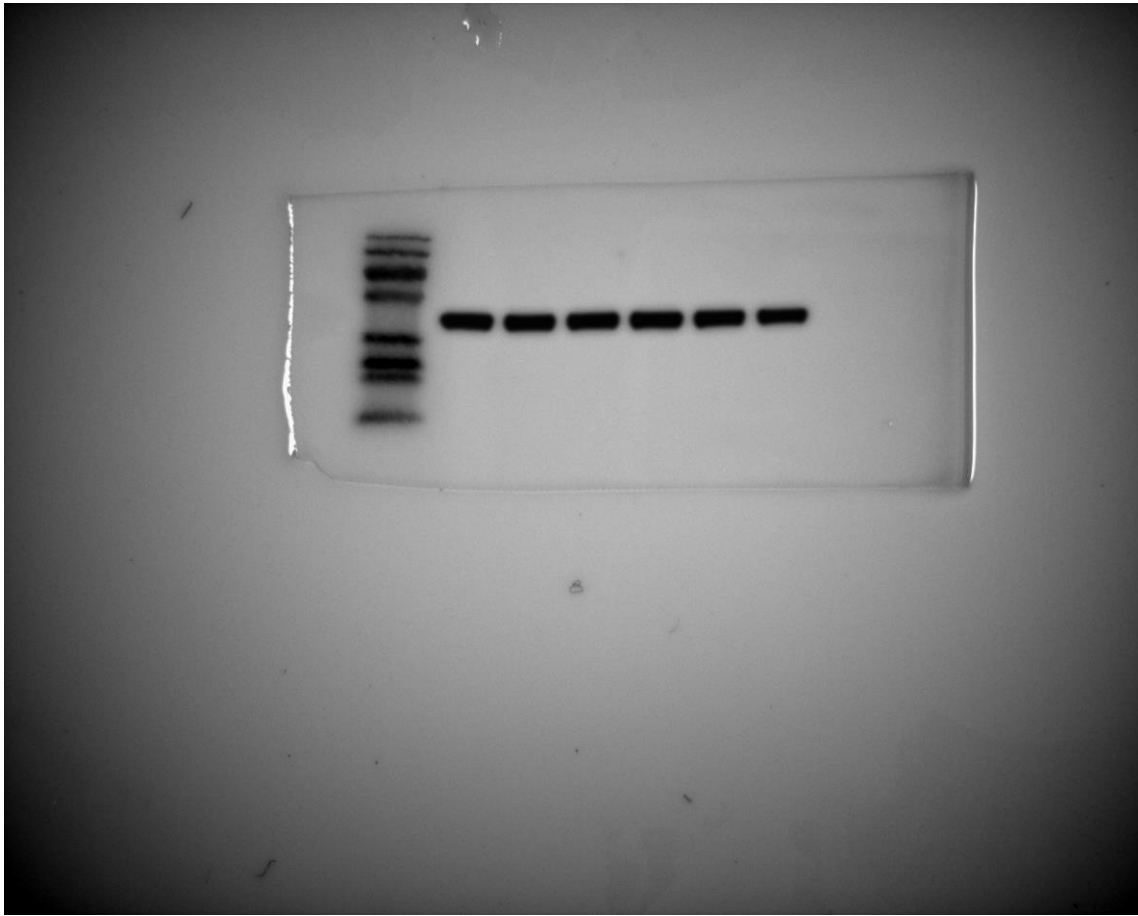

Figure 6A GAPDH

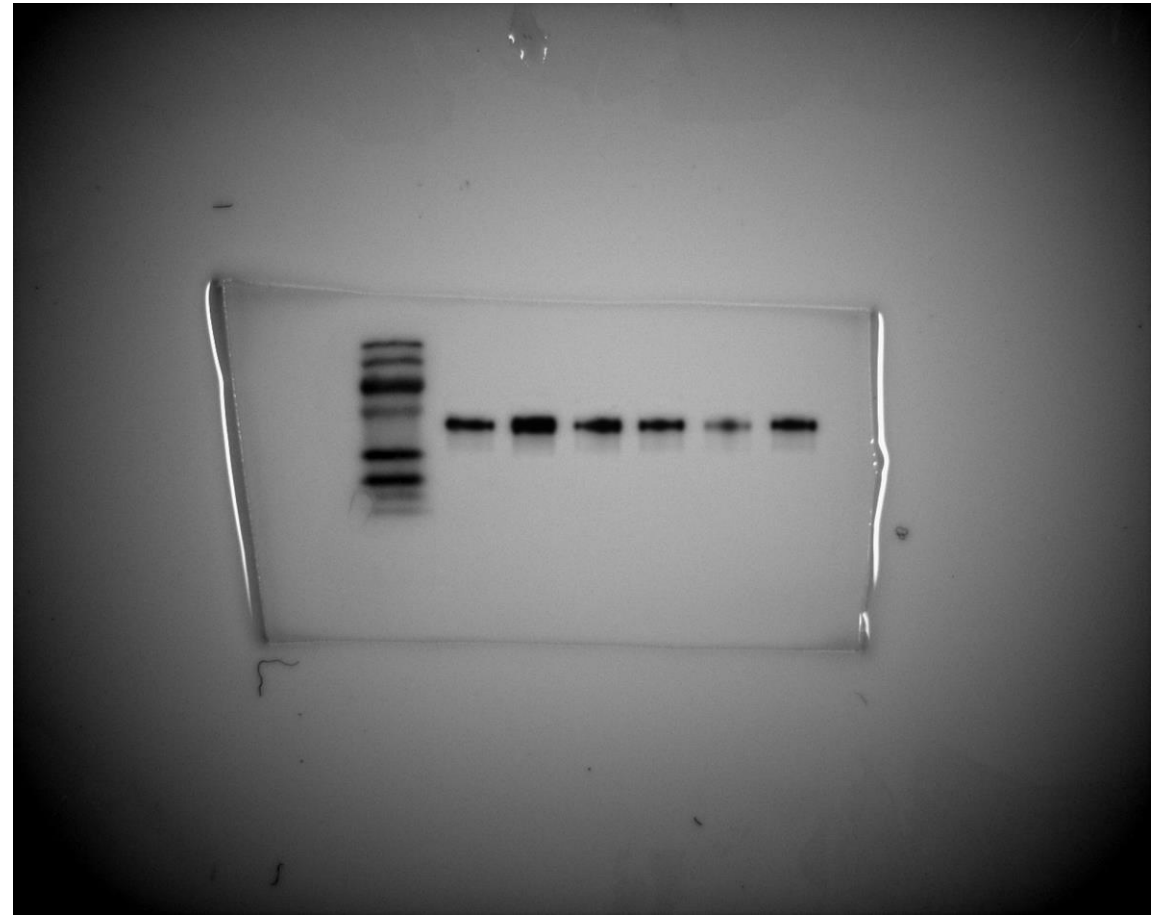

Figure 6A PTMA

Supplement: Supplementary file 9 — Supplementary Figure 7. [file 41598_2024_52578_MOESM9_ESM.pdf]

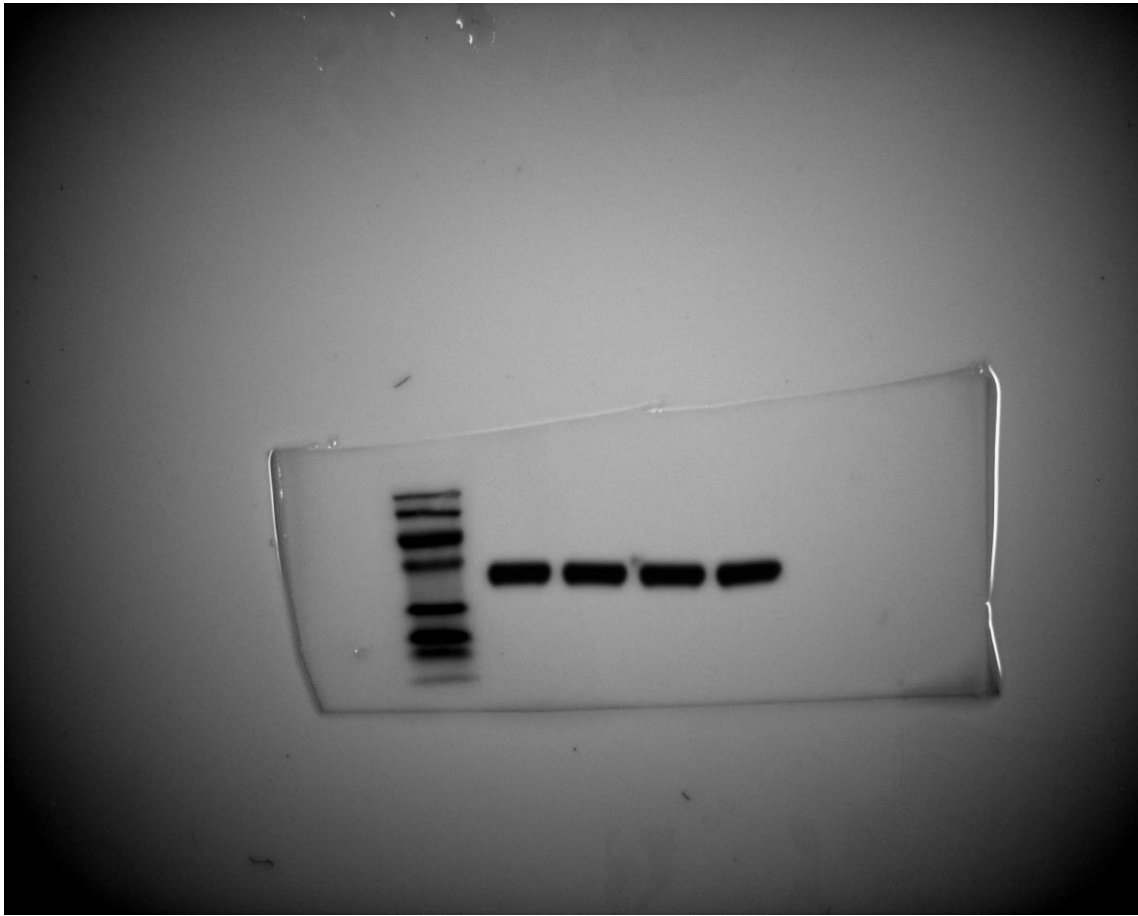

Figure 7D GAPDH

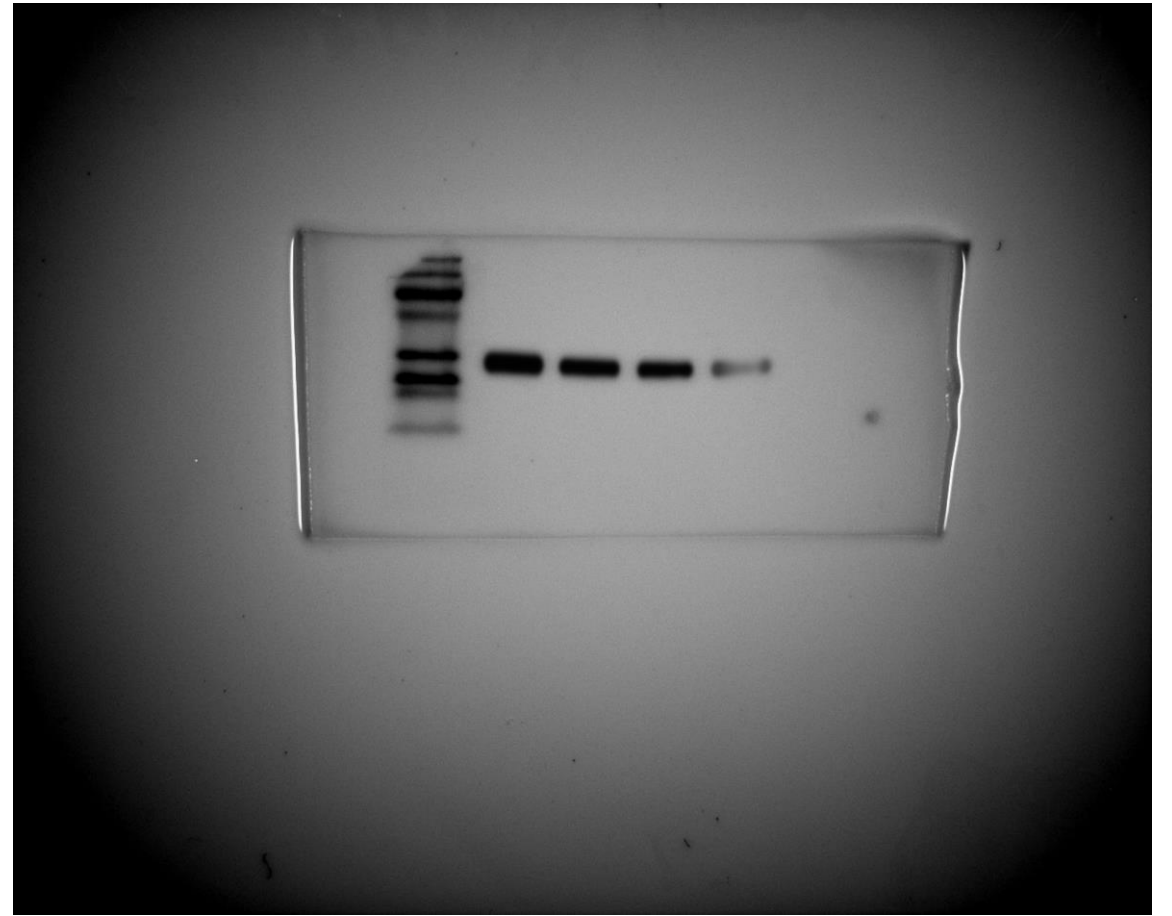

Figure 7D PTMA

Supplement: Supplementary file 10 — Supplementary Figure 3. [file 41598_2024_52578_MOESM10_ESM.pdf]
